# Gut microbiota-regulated cell death: a review on pyroptosis, ferroptosis, and related mechanisms

**DOI:** 10.3389/fmicb.2026.1853772

**Published:** 2026-08-26

**Authors:** Yi Shen, Yunfeng Zhou, Yang Yuan, Wei Chen, Min Liang, Xiong Liu, Yusong Lu, Qiangqiang Zheng

**Affiliations:** Department of Thoracic Surgery, West China School of Public Health and West China Fourth Hospital, Sichuan University, Chengdu, Sichuan, China

**Keywords:** disease mechanisms, ferroptosis, inflammation, intestinal microbiota, metabolic disorder, programmed cell death, pyroptosis

## Abstract

The intestinal microbiota has emerged as a crucial regulator of host physiological functions, significantly influencing various forms of regulated cell death (RCD). Among these, pyroptosis and ferroptosis play pivotal roles in immune regulation, metabolic homeostasis, and disease progression. The gut microbiota modulates these cell-death pathways through microbial metabolites, inflammatory signaling, oxidative stress, and immune-cell polarization, thereby affecting intestinal disorders, metabolic diseases, cardiovascular diseases, and neurodegenerative diseases. However, current evidence remains fragmented across individual cell-death pathways, and direct microbiota-mediated regulation is often not clearly distinguished from indirect associations arising from barrier dysfunction, inflammation, or metabolic remodeling. This review critically synthesizes the regulatory networks linking the gut microbiota to pyroptosis, ferroptosis, and related cell-death mechanisms. Throughout, we use RCD as the umbrella term for genetically encoded death programs and reserve programmed cell death (PCD) for its physiological, developmental subset, and we grade the supporting evidence at four levels: direct microbial regulation of the death machinery, experimentally demonstrated microbiota-dependent regulation, indirect effects mediated by barrier dysfunction or inflammation, and purely observational association. By integrating recent findings, we discuss the potential and limitations of therapeutic strategies targeting microbiota-regulated cell death. This synthesis provides a framework for defining current evidence gaps and guiding future mechanistic and translational studies.

## 1 Introduction

Programmed cell death (PCD) represents a fundamental biological process essential for maintaining organismal homeostasis and regulating immune responses. Terminology in this field is used inconsistently, and we therefore adopt the conventions of the Nomenclature Committee on Cell Death. Regulated cell death (RCD) denotes any genetically encoded, molecularly controlled death program that can be modulated pharmacologically or genetically, whereas PCD is restricted to the physiological subset of RCD that occurs during development and normal tissue turnover (Galluzzi et al., 2018). Pyroptosis, ferroptosis, necroptosis, cuproptosis and disulfidptosis are accordingly referred to as RCD modalities throughout this review, and PCD is retained only where the original studies described physiological cell turnover. Beyond the classical apoptosis, recent advances have identified novel forms of RCD, including pyroptosis and ferroptosis, which have garnered significant attention due to their unique molecular mechanisms and implications in physiology and pathology. Pyroptosis is characterized as a lytic, inflammatory form of cell death mediated by gasdermin (GSDM) family proteins, leading to membrane pore formation, cell swelling, and release of pro-inflammatory intracellular contents. This process plays a pivotal role in host defense against infections and modulates immune responses (Patton et al., 2026). Ferroptosis, on the other hand, is an iron-dependent form of regulated necrosis driven by overwhelming lipid peroxidation and reactive oxygen species (ROS) accumulation, distinct from apoptosis and necroptosis (Yao and Li, 2023). Both pyroptosis and ferroptosis contribute to inflammatory signaling and immune regulation, underscoring their importance in disease pathogenesis and therapeutic targeting (Chang et al., 2024; Li and Jiang, 2025). Understanding the molecular pathways governing these cell-death forms has opened new avenues for intervention in diverse diseases, including cancer, neurodegeneration, and inflammatory disorders (Tang et al., 2020; Wang Q. et al., 2023).

The gut microbiota, comprising trillions of microorganisms residing in the intestinal tract, forms a complex and dynamic ecosystem that profoundly influences host metabolism, immunity, and cellular functions (Al-Rashidi, 2022; Ezenabor et al., 2024). This symbiotic microbial community participates in nutrient metabolism, production of bioactive metabolites, and modulation of mucosal and systemic immune responses. Disturbances in gut microbial composition, termed dysbiosis, have been implicated in a wide spectrum of diseases, including metabolic syndrome, inflammatory bowel disease (IBD), neurodegenerative disorders, and cancers (Ezenabor et al., 2024; Sharma et al., 2023; Sultan S. et al., 2021). The crosstalk between gut microbiota and host immunity is bidirectional: while the microbiota shapes immune cell development and function, the immune system maintains microbial homeostasis and barrier integrity (Brown et al., 2025; Holmes et al., 2020). Notably, microbial metabolites such as short-chain fatty acids (SCFAs), bile acids, and tryptophan derivatives modulate immune signaling pathways, influencing inflammation and tissue homeostasis (Pei et al., 2025; Zhao Q. et al., 2023). Dysbiosis can disrupt these interactions, leading to aberrant immune activation, impaired barrier function, and altered cellular processes, thereby contributing to disease progression (Shen et al., 2025b; Zhao M. et al., 2023).

Emerging evidence highlights that the gut microbiota has been linked to RCD pathways, including pyroptosis and ferroptosis, thereby influencing disease outcomes (Yao and Li, 2023; Zhang and Qin, 2026). The microbiota and its metabolites can modulate inflammatory cell death by affecting immune cell activation, cytokine production, and oxidative stress (Bao et al., 2024; Huang et al., 2024). For instance, microbial-derived metabolites such as aryl hydrocarbon receptor (AhR) ligands can inhibit ferroptosis in intestinal intraepithelial lymphocytes, impacting mucosal immunity and inflammation (Liu R. et al., 2024). Conversely, dysbiosis-associated pathogenic bacteria may promote pyroptosis and ferroptosis through toxin release and metabolic disturbances, exacerbating tissue injury (Fang et al., 2024; Zhang D. et al., 2022). In IBD, the interplay between gut microbiota, pyroptosis, and ferroptosis contributes to epithelial barrier disruption and chronic inflammation (Bao et al., 2024; Liu A. et al., 2025). Moreover, modulation of gut microbiota has been shown to influence immune checkpoint pathways such as programmed death-1 (PD-1)/programmed death-ligand 1 (PD-L1), which are critical regulators of immune tolerance and antitumor immunity (He et al., 2022; Zhou et al., 2022). The gut microbiota’s role in regulating these immune checkpoints further underscores its impact on RCD and immune responses (Liu et al., 2022; Souffriau et al., 2020).

Collectively, the gut microbiota functions as a key regulator of pyroptosis and ferroptosis through complex molecular networks involving immune modulation, metabolic signaling, and cellular stress responses. Dysbiosis disrupts these regulatory circuits, promoting aberrant cell death and inflammation, thereby influencing the progression of multiple diseases. Understanding the molecular interplay between gut microbiota and RCD pathways holds promise for developing novel therapeutic strategies targeting microbiota composition and function to restore immune homeostasis and mitigate disease (Huang et al., 2024; Yao and Li, 2023; Zhang and Qin, 2026). This review aims to provide a comprehensive discussion of the gut microbiota’s regulation of pyroptosis and ferroptosis, elucidate the underlying molecular mechanisms, and explore their implications in disease pathophysiology and treatment.

Because the studies integrated here differ widely in experimental depth, two interpretive conventions are applied throughout. The first concerns how pyroptosis and ferroptosis are identified. Neither modality can be established from a single biomarker. Altered expression of NLRP3, caspase-1, GSDMD, GPX4, ACSL4 or SLC7A11, or changes in bulk ROS or malondialdehyde levels, indicate that a pathway has been perturbed but do not demonstrate that cells actually died through that pathway; such markers also change during sublethal stress, during apoptosis and secondary necrosis, and as a consequence rather than a cause of tissue injury. Current nomenclature recommendations require that pyroptosis be supported by gasdermin cleavage together with gasdermin-N-dependent membrane permeabilization, by lytic death measured with an independent assay such as lactate dehydrogenase release or propidium iodide uptake, by IL-1β/IL-18 maturation and release, and critically by rescue upon genetic deletion of GSDMD or GSDME or upon caspase-1/4/5/11 inhibition (Galluzzi et al., 2018; Shi et al., 2015). Ferroptosis similarly requires evidence of iron-dependent phospholipid peroxidation, for example oxidized phosphatidylethanolamine species or C11-BODIPY oxidation, together with rescue by lipophilic radical-trapping antioxidants such as ferrostatin-1 or liproxstatin-1, by iron chelation or by ACSL4 deletion, and the absence of rescue by caspase or necroptosis inhibitors (Galluzzi et al., 2018; Hadian and Stockwell, 2020; Tang and Kroemer, 2020). A substantial proportion of the microbiota-related studies summarized below report marker changes only. Where this is the case, the findings should be read as evidence of pathway engagement rather than as proof of pyroptotic or ferroptotic execution, and we have attempted to signal this limitation in the text and in Tables 1, 2.

**Table 1:** Gut microbiota–regulated pyroptosis: molecular mechanisms, microbial factors, and disease relevance.

Microbial role	Microbial factor/metabolite	Host signaling pathway	Key pyroptosis regulators	Affected cell type(s)	Disease context	Functional outcome	Therapeutic implications (specific intervention/experimental model)	Reference(s)
Pro-inflammatory (pathogenic)	LPS from Gram-negative bacteria/dysbiosis-associated endotoxemia	TLR4 → NF-κB → NLRP3 inflammasome priming and canonical caspase-1 activation	Caspase-1, GSDMD, IL-1β, IL-18	IECs, macrophages	IBD, metabolic syndrome, liver fibrosis	Promotes epithelial/macrophage pyroptosis, disrupts barrier integrity, amplifies systemic inflammation	Barrier-protective probiotic (*L. acidophilus* HSCC LA042) suppressed NLRP3-dependent pyroptosis in a DSS-induced colitis mouse model	Zhu et al. (2024)
Pro-inflammatory (pathogenic)	Cytosolic LPS from Gram-negative pathobionts (noncanonical entry)	Caspase-4/5 (human)/caspase-11 (mouse) direct LPS sensing → GSDMD cleavage → K + efflux → secondary NLRP3 activation	Caspase-4/5/11, GSDMD, NLRP3, IL-1β/IL-18	IECs, macrophages	Barrier-disrupted gut, pathobiont invasion, sepsis	Forms membrane pores independent of canonical priming; amplifies IL-1β/IL-18 maturation	Noncanonical caspase inhibition or limiting pathobiont/outer-membrane-vesicle translocation; mechanism established in murine cytosolic-LPS transfection models	Shi et al. (2014), Rühl and Broz (2015), and Shi et al. (2015)
Pro-inflammatory (pathogenic)	*Peptostreptococcus anaerobius* (dysbiosis-associated pathobiont)	TLR2/4 → NF-κB → NLRP3	Caspase-1, GSDMD	Macrophages	Colitis, IBD	Exacerbates macrophage pyroptosis and intestinal barrier disruption	Pathobiont-targeted decolonization/TLR2/4 blockade; demonstrated in a DSS-induced colitis mouse model	Shen et al. (2024)
Pro-inflammatory (pathogenic)	MCT4-linked microbiota-associated metabolic signaling	ERK1/2 → NF-κB → caspase-1 activation	Caspase-1, MCT4	IECs	IBD	Aggravates intestinal inflammation via IEC pyroptosis	MCT4 inhibition proposed as an adjunct anti-pyroptotic strategy; supported by IBD patient tissue and in vitro IEC data	Wang et al. (2021)
Pro-inflammatory (pathogenic)	Gut dysbiosis-driven systemic signaling	Dysbiosis → GSDME-mediated macrophage pyroptosis	GSDME, caspase-3	Macrophages, ovarian granulosa cells	Polycystic ovary syndrome	Disrupts steroidogenesis and promotes granulosa-cell apoptosis via systemic macrophage pyroptosis	Microbiota restoration to limit GSDME-driven macrophage pyroptosis; shown in a PCOS mouse model	Huang et al. (2022)
Pro-inflammatory (pathogenic)	TMAO (choline/L-carnitine–derived metabolite)	mROS–NLRP3 axis	Caspase-1, NLRP3	Renal tubular cells, endothelial cells	Diabetic kidney disease, T2D vascular complications	Drives pyroptosis-mediated renal and vascular injury	TCM formula Zuogui-Jiangtang-Yishen decoction suppressed TMAO-induced mROS-NLRP3 pyroptosis in a diabetic kidney disease rat model	Yi et al. (2023) and Zhang and Qin (2026)
Pro-inflammatory (pathogenic)	Arsenic-altered gut microbiota	AhR/NF-κB/NLRP3 axis; increased intestinal permeability	Caspase-1, NLRP3	Microglia	Arsenic-induced Alzheimer’s disease-like pathology	Drives microglial pyroptosis and neuroinflammation via the gut-brain axis	Reducing arsenic-induced dysbiosis/permeability; demonstrated in a chronic arsenic-exposure mouse model	Qu et al. (2026)
Beneficial (protective)	SCFAs (butyrate, propionate)	HDAC inhibition; suppression of NLRP3 priming and assembly	Reduced caspase-1 cleavage, inhibited GSDMD pore formation	IECs, macrophages	IBD, piglet diarrhea, cardiac inflammation	Suppresses pyroptosis and IL-1β/IL-18 release	FMT that raised fecal butyrate reduced LPS-induced oxidative stress/pyroptosis in a piglet diarrhea model; hydroxysafflor yellow A suppressed HK1/NLRP3/GSDMD-mediated colitis pyroptosis in mice	Liu M. et al. (2024) and Chen J. et al. (2023)
Beneficial (protective)	Probiotic strain *E. coli* Nissle 1917	Homocitrulline-mediated suppression of the caspase-1/NLRP3/GSDMD axis	Caspase-1, NLRP3, GSDMD (downregulated)	Macrophages, vascular tissue	Atherosclerosis, sepsis	Attenuates pyroptosis and systemic inflammation	Oral *E. coli* Nissle 1917 supplementation reduced atherosclerotic pyroptosis via serum homocitrulline modulation in ApoE−/− mice	Liu H. et al. (2025)
Beneficial (protective)	Xanthohumol (microbiota-derived metabolite)	Suppression of NF-κB-dependent transcription	Reduced NLRP3, caspase-1, GSDMD	Macrophages	Heatstroke, systemic inflammation	Inhibits macrophage pyroptosis, limits systemic inflammatory injury	Xanthohumol administration protected against heatstroke-induced macrophage pyroptosis in a murine heatstroke model	Huang et al. (2026)
Context-dependent	TCM-modulated microbiota (e.g., modified Gexia-Zhuyu Tang)	Multi-pathway regulation of NF-κB/inflammasome signaling	Caspase-1, GSDMD	IECs, gastric tumor cells	IBD, gastric cancer	Suppresses pyroptosis in inflamed intestinal epithelium but promotes tumor-cell pyroptosis in gastric cancer	Modified Gexia-Zhuyu Tang restored gut microbial diversity while promoting caspase-1-dependent tumor pyroptosis in a gastric cancer mouse model	Zhao and Yu (2024)

**Table 2:** Gut microbiota–ferroptosis axis: metabolic regulation, molecular targets, and disease implications.

Microbial role	Microbial component/metabolite	Regulatory mechanism	Core ferroptosis molecules	Target tissue(s)/cell(s)	Associated disease(s)	Biological consequence	Therapeutic opportunities (specific intervention/experimental model)	Reference(s)
Pro-ferroptotic (harmful)	Dysbiosis-induced iron metabolism disorder	Increased intestinal iron absorption and hepatic/neuronal iron storage	TFR1, FPN1, ACSL4	Hepatocytes, neurons	Liver injury, neurodegeneration	Promotes iron overload and lipid peroxidation, sensitizing cells to ferroptosis	Correction of iron homeostasis via microbiota remodeling (FMT/synbiotics); supported by a liver-specific Fbxl5-null mouse model of iron-overload liver injury	Fan et al. (2022) and Matsumoto et al. (2025)
Pro-ferroptotic (harmful)	LPS (indirect effect via inflammatory ROS amplification)	Inflammatory ROS amplification suppressing GPX4	GPX4 (suppressed), lipid ROS	IECs, immune cells	IBD, metabolic inflammation	Sensitizes epithelial and immune cells to ferroptosis	Anti-inflammatory/barrier-restoring microbiota interventions to limit LPS-driven ROS; supported by IBD-dysbiosis ferroptosis studies	Yao and Li (2023)
Pro-ferroptotic (harmful)	TMAO	Destabilization of the OTUB1/SLC7A11 axis	SLC7A11 (destabilized), GPX4 (reduced)	Hepatocytes	MASLD	Promotes hepatocyte ferroptosis, worsens hepatic injury	TMAO-lowering microbiota interventions (probiotics/FMT) to preserve OTUB1/SLC7A11 stability; described in gut-liver MASLD mechanistic studies	Zhuge et al. (2025) and Ji et al. (2023)
Pro-ferroptotic (harmful)	Heavy metal (nickel, arsenic)-altered microbiota	ROS accumulation and lipid peroxidation	ACSL4 (upregulated), GPX4 (inhibited)	Hepatocytes, neurons	Heavy metal-induced liver and neuro injury	Triggers ferroptotic hepatocyte/neuronal death via gut-liver and gut-brain axes	FMT plus ferroptosis inhibitors (e.g., ferrostatin-1) or *Faecalibacterium prausnitzii* supplementation; shown in nickel- and arsenic-exposure mouse models	Shen et al. (2025a), Shao et al. (2024), and Zhang Y. et al. (2025)
Pro-ferroptotic (harmful)	Dysbiosis-associated NOX4 upregulation	NADPH oxidase-driven ROS generation exacerbating lipid peroxidation	NOX4, GPX4 (suppressed), FSP1 (protective target)	Dopaminergic neurons	Parkinson’s disease	Promotes neuronal ferroptosis and neuroinflammation	NOX4 inhibition or FSP1/GPX4-targeted neuroprotection; demonstrated in Parkinson’s disease models	Lin et al. (2025) and Chen and Xie (2020)
Beneficial (protective)	SCFAs (butyrate, valerate)	GPR43/GPR109A signaling and HDAC inhibition enhancing Nrf2/GPX4 antioxidant defense	GPX4 (upregulated), Nrf2 activation	Neurons, intestinal cells	Neuroinflammation, stress-related disorders	Suppresses ferroptosis and oxidative injury via the gut-brain axis	Butyrate/valerate supplementation attenuated stress-induced hippocampal and prefrontal-cortex ferroptosis in mouse models	Ma X. et al. (2025) and Wang Z. et al. (2025)
Beneficial (protective)	Bile acid metabolites	FXR/TGR5/S1PR2-mediated redox regulation	SLC7A11, GPX4 (upregulated)	Hepatocytes	MASLD, liver fibrosis	Restores redox balance and ferroptosis resistance	Microbiota-targeted bile-acid-pool modulation; supported by gut-liver MASLD mechanistic reviews	Ma H. et al. (2025) and Termite et al. (2025)
Beneficial (protective)	AhR ligands (tryptophan-derived microbial metabolites)	AhR signaling inhibits ferroptosis	GPX4-associated antioxidant defense	Intestinal intraepithelial lymphocytes	Mucosal immunity, tumor immunotherapy	Preserves intraepithelial lymphocyte viability, supporting mucosal and antitumor immunity	AhR ligand supplementation proposed as an adjunct to tumor immunotherapy to protect immune cells from ferroptosis; preclinical immune-cell ferroptosis studies	Liu R. et al. (2024)
Beneficial (protective)	Capsiate (microbiota-dependent metabolite)	TRPV1 activation → GPX4 upregulation	GPX4 (stabilized)	IECs, vascular endothelium	Intestinal ischemia/reperfusion injury, atherosclerosis	Inhibits ferroptosis-induced epithelial and vascular damage	Capsiate reduced ferroptosis in intestinal I/R injury and reshaped gut microbiota to attenuate atherosclerosis in ApoE−/− mice via Nrf2/GPX4	Deng et al. (2021) and Shen et al. (2025c)
Beneficial (protective)	*Akkermansia muciniphila*–derived acetate	Hepatic AMPK/SIRT1/PGC-1α axis activation	GPX4 (preserved), mitochondrial function restored	Hepatocytes	MASLD	Alleviates hepatocyte ferroptosis and improves mitochondrial function	*A. muciniphila* (strain Akk11) combined with a GLP-1 receptor agonist reduced adiposity and hepatic ferroptosis in T2D/MASLD mouse models	Zhuge et al. (2025) and Gao et al. (2026)
Beneficial (protective)	Probiotic *Faecalibacterium prausnitzii*	Butyrate-mediated LCN2 regulation	GPX4 (preserved)	Cardiomyocytes, liver, gut	Age-related heart failure, liver fibrosis, colitis	Attenuates ferroptosis and tissue injury	*F. prausnitzii* supplementation prevented age-related heart failure by suppressing cardiomyocyte ferroptosis via butyrate-LCN2 signaling in aged mice	Zhang J. et al. (2025)
Beneficial (protective)	Natural compounds via microbiota modulation (baicalin, ginsenoside Rd)	Redox homeostasis restoration	SLC7A11, GPX4 (up); ACSL4, ROS (down)	Neurons, hepatocytes	Neurodegeneration/depression, MASLD	Inhibits ferroptosis and promotes functional recovery	Baicalin improved depressive behavior by inhibiting nerve-cell ferroptosis in atherosclerotic mice; ginsenoside Rd. modulated the gut microbiota-ferroptosis pathway in a MASLD mouse model	Ren et al. (2025) and Liu W. et al. (2025)

The second convention concerns the strength of the microbiota–cell death link, which we grade at four levels. Level 1, direct microbial regulation, applies when a defined microbial component or metabolite acts on an identified host sensor, receptor or enzyme to alter the death machinery, as in cytosolic lipopolysaccharide (LPS) sensing by caspase-4/5/11 or short-chain fatty acid (SCFA) engagement of GPR43/GPR109A and histone deacetylases. Level 2, experimentally demonstrated microbiota-dependent regulation, applies when causality is supported by germ-free or gnotobiotic colonization, antibiotic depletion with reconstitution, fecal microbiota transfer, or mono-colonization with a defined strain, combined with a cell-death readout. Studies satisfying both levels are still uncommon; a recent example is the demonstration that Ligilactobacillus salivarius-derived indole-3-lactic acid (ILA) drives terminal exhaustion of tumor-infiltrating NKG7 + CD8 + T cells through AhR-dependent suppression of NF-κB signaling, in which the responsible strain, the metabolite, the host receptor and the downstream immune outcome were each identified, isogenic ILA-deficient strains were used to establish necessity, and the mechanism was tested in humanized-microbiome models and two independent patient cohorts (Zhou et al., 2026). Level 3, indirect regulation, applies when the microbiota alters barrier permeability, systemic inflammation, bile-acid pools, iron distribution or immune-cell polarization, and cell death changes secondarily. Level 4, association, applies when microbiota composition and cell-death markers are measured in parallel in patients or animals without any intervention. Most human data in this field currently remain at level 4, and a large part of the animal literature at level 3. Accordingly, causal verbs such as “induces,” “drives” or “mediates” are used only for level 1 and level 2 findings, whereas “is associated with,” “correlates with” or “may contribute to” are used for level 3 and level 4 findings. Human studies are summarized separately in Table 3, which lists population, sample size, study design, microbiome method, cell-death readout, tissue assessed, intervention and clinical outcome for each report, so that human evidence can be appraised independently of the cell-culture and animal literature summarized in Tables 1, 2.

**Table 3:** Human studies relevant to gut microbiota-regulated cell death: populations, designs, microbiome methods, cell-death readouts and clinical outcomes.

Population	Sample size	Study design	Microbiome method	Cell-death markers assessed	Tissue assessed	Intervention	Clinical outcome	Reference(s)
Patients with locally advanced ESCC receiving neoadjuvant anti-PD-1 therapy	122 fecal samples (responders vs. non-responders)	Prospective observational cohorts (two independent) with mechanistic validation in humanized-microbiome and orthotopic mouse models	Fecal microbiota profiling with targeted metabolomics (ILA) and single-cell RNA sequencing of tumor tissue	No direct RCD readout; immune-effector and exhaustion phenotype (NKG7 + CD8 + Tpex cells, AhR-NF-κB signaling)	Feces; tumor tissue	Neoadjuvant anti-PD-1 (standard of care); no microbiota intervention in patients	Enrichment of *L. salivarius* and its metabolite ILA associated with non-response to anti-PD-1; causality shown only in mice	Zhou et al. (2026)
Previously untreated patients with advanced cutaneous melanoma	20	Phase I, single-arm, multicenter (MIMic)	Shotgun metagenomic sequencing of donor and recipient stool	Not assessed	Feces; peripheral blood	Healthy-donor FMT (oral capsules) plus nivolumab or pembrolizumab	Safe and feasible; objective response rate 65% (13/20), including 4 complete responses	Routy et al. (2023)
Patients with advanced NSCLC or cutaneous/uveal melanoma starting first-line immune checkpoint blockade	40 (NSCLC *n* = 20; melanoma *n* = 20)	Phase 2, single-arm, multicenter (FMT-LUMINate, NCT04951583)	Shotgun metagenomic sequencing of donor and recipient stool	Not assessed	Feces; peripheral blood	Single healthy-donor oral-capsule FMT 1 week before first-line ICI	Objective response rate 80% (NSCLC) and 75% (melanoma); response associated with loss of baseline commensal species rather than donor engraftment	Duttagupta et al. (2026)
Adults with recurrent Clostridioides difficile infection	182 randomized	Phase 3, double-blind, placebo-controlled randomized trial (ECOSPOR III)	16S rRNA gene and metagenomic profiling of stool	Not assessed	Feces	SER-109 (oral consortium of purified Firmicutes spores) vs. placebo	Reduced recurrence at 8 weeks versus placebo (12% vs. 40%)	Feuerstadt et al. (2022)
Patients undergoing elective diagnostic coronary angiography	4,007 (plus a small antibiotic-suppression sub-study)	Prospective observational cohort with human interventional sub-study	Plasma TMAO quantification; microbial dependence established by suppression and recovery of TMAO after oral antibiotics	Not assessed	Plasma	None (observational); oral broad-spectrum antibiotics in the sub-study	Elevated plasma TMAO independently associated with 3-year risk of major adverse cardiovascular events	Koeth et al. (2013) and Tang et al. (2013)
Population-based GWAS datasets for gut microbial taxa and ARDS	Summary-level GWAS data (no individual participants)	Two-sample Mendelian randomization	16S rRNA-based microbial GWAS summary statistics	Not assessed	Not applicable	None (genetic instrument analysis)	Specific taxa (Streptococcus, Bifidobacterium, Akkermansia) associated with ARDS risk or severity; causality not established	Huang et al. (2025) and Li J. et al. (2025)

## 2 Regulatory mechanisms of gut microbiota and pyroptosis

### 2.1 Molecular mechanisms of pyroptosis and its physiological and pathological significance

Pyroptosis is a lytic and inflammatory form of regulated cell death, fundamentally differing from apoptosis. It is primarily mediated by the GSDM protein family, especially gasdermin D (GSDMD), which acts as the executioner molecule forming membrane pores that lead to cell swelling and eventual rupture. The initiation of pyroptosis hinges on the activation of inflammasomes—multiprotein complexes such as the NOD-like receptor family pyrin domain containing 3 (NLRP3) inflammasome—that sense pathogenic or danger signals. Upon activation, inflammasomes recruit and activate inflammatory caspases, notably caspase-1 in the canonical pathway and caspase-4/5/11 in the non-canonical pathway. These caspases cleave GSDMD to release its N-terminal domain, which oligomerizes and inserts into the plasma membrane to form pores, facilitating the release of pro-inflammatory cytokines interleukin-1 beta (IL-1β) and interleukin-18 (IL-18) and resulting in a potent inflammatory response (Burdette et al., 2021; Liu and Lieberman, 2024; Yang et al., 2022). In addition to NLRP3, NLRP1 can respond to pathogen-derived protease activity and cellular stress; NLRC4 is activated by bacterial flagellin and type III or type IV secretion system components; AIM2 senses cytosolic double-stranded DNA from host damage or microbial infection; and pyrin detects bacterial toxin-mediated Rho GTPase inactivation. This inflammatory cell death serves as a critical component of innate immunity by eliminating intracellular pathogens and alerting neighboring cells to infection or damage, thus initiating and amplifying immune responses (Sollberger, 2022). Pyroptosis is not confined to immune cells such as macrophages and monocytes but has also been observed in epithelial cells and other cell types, highlighting its broad physiological relevance (Johnson and Cui, 2025; Liu and Lieberman, 2024).

The noncanonical pyroptosis pathway further expands this microbial sensing network (Shi et al., 2014). In humans, caspase-4 and caspase-5, and in mice, caspase-11, directly sense cytosolic LPS derived from Gram-negative bacteria. Activated caspase-4/5/11 cleaves GSDMD to induce pyroptotic membrane pore formation and potassium efflux, which can secondarily activate the NLRP3 inflammasome and promote IL-1β/IL-18 maturation (Rühl and Broz, 2015; Shi et al., 2015). This pathway is highly relevant to gut microbiota because epithelial barrier disruption, intracellular invasion by Gram-negative pathobionts, bacterial outer membrane vesicles, or LPS delivery into the cytosol may activate noncanonical pyroptosis even when canonical inflammasome priming is incomplete.

In the gastrointestinal tract, pyroptosis plays a dual role. Physiologically, it contributes to gut homeostasis by mediating the clearance of pathogens and damaged cells, thereby maintaining the integrity of the intestinal barrier. GSDMs are expressed in intestinal epithelial cells (IECs) and lamina propria immune cells, where their activation leads to the removal of self-danger molecules and pathogen niches through inflammatory cell death (Gong et al., 2022). However, dysregulated or excessive pyroptosis is implicated in pathological conditions, particularly in intestinal barrier damage and inflammatory diseases such as IBD and ulcerative colitis (UC). The release of intracellular contents and inflammatory cytokines following pyroptotic cell death exacerbates mucosal inflammation, disrupts epithelial tight junctions, and promotes intestinal permeability, thereby perpetuating a cycle of inflammation and tissue injury (Al Mamun et al., 2021; Bao et al., 2024). For instance, activation of the NLRP3 inflammasome and subsequent pyroptosis in intestinal macrophages and epithelial cells have been shown to drive the pathogenesis of colitis, with therapeutic interventions targeting these pathways demonstrating efficacy in experimental models (Bao et al., 2024; Zhu et al., 2024).

In summary, pyroptosis is a molecularly defined, caspase-dependent inflammatory cell death pathway orchestrated by inflammasome activation and GSDM-mediated membrane pore formation. It plays a vital physiological role in immune defense and tissue homeostasis, particularly within the gut. However, its aberrant activation contributes to intestinal barrier injury and the pathogenesis of inflammatory diseases, highlighting pyroptosis as a critical molecular nexus linking innate immunity, inflammation, and tissue pathology in the gastrointestinal system (Bao et al., 2024; Gong et al., 2022; Liu and Lieberman, 2024; Yang et al., 2022).

### 2.2 Regulatory pathways of gut microbiota on pyroptosis

The gut microbiota exerts a profound influence on pyroptosis, through multiple regulatory pathways that integrate microbial components, metabolites, and host barrier interactions. Building on the core pyroptotic machinery described above, microbiota-mediated regulation may occur either directly, through intracellular microbial components that activate inflammasomes or inflammatory caspases, or indirectly, through changes in intestinal barrier integrity, inflammatory signaling, and metabolite availability. One shared entry point involves dysbiosis-associated microbial products, particularly LPS, which can activate TLR-dependent NF-κB signaling and provide the priming signal for NLRP3 inflammasome activation. Barrier impairment may further facilitate the translocation of LPS and other microbial products, thereby indirectly lowering the threshold for pyroptotic activation in intestinal and extraintestinal tissues. However, this LPS-centered pathway should be interpreted as a common upstream trigger rather than a disease-specific mechanism. For instance, in IBD and colitis, pathogenic bacteria such as *Peptostreptococcus anaerobius* exacerbate inflammation by activating TLR2/4–NF-κB–NLRP3 signaling, promoting macrophage pyroptosis and intestinal barrier disruption (Shen et al., 2024).

Beyond endotoxin-driven inflammasome activation, microbial metabolites critically modulate pyroptosis. SCFAs, primarily produced by fermentation of dietary fibers by commensal bacteria, particularly butyrate, may suppress inflammasome activation by regulating mitochondrial stress, epithelial metabolism and inflammatory transcriptional programs. For example, fecal microbiota transplantation (FMT) that increases butyric acid levels attenuates LPS-induced oxidative stress and pyroptosis, improving intestinal barrier integrity and reducing diarrhea in piglets (Liu M. et al., 2024). Conversely, dysbiotic microbiota may reduce beneficial SCFA production, favoring pyroptosis and inflammation, as observed in UC where altered microbial communities correlate with increased pyroptotic markers (Chen J. et al., 2023; Zhang J. et al., 2022). Nevertheless, the effects of SCFAs may vary according to their concentration, target cell type, and disease context and should not be regarded as uniformly protective.

Furthermore, the crosstalk between gut microbiota and host immune cells involves complex signaling pathways. Probiotic strains such as *Escherichia coli Nissle 1917* can modulate gut microbiota composition and serum metabolites to counteract pyroptosis via the homocitrulline/Caspase 1/NLRP3/GSDMD axis, highlighting the therapeutic potential of microbiota modulation (Liu H. et al., 2025). Additionally, gut microbiota-derived metabolites such as xanthohumol inhibit macrophage pyroptosis by suppressing nuclear factors transcription, thereby protecting against systemic inflammation in heatstroke (Huang et al., 2026). Traditional Chinese medicine (TCM) formulations also demonstrate the ability to regulate gut microbiota and pyroptosis pathways, exemplified by modified Gexia-Zhuyu Tang promoting caspase-1-dependent pyroptosis in gastric cancer while restoring microbial diversity (Zhao and Yu, 2024). To provide an integrated overview of microbial factors, host signaling pathways, and disease contexts involved in microbiota-regulated pyroptosis, the key mechanisms are summarized in Table 1.

### 2.3 Role of pyroptosis in diseases and microbial regulation

IBD, encompassing Crohn’s disease (CD) and UC, is characterized by chronic intestinal inflammation with complex pathogenesis involving genetic, environmental, immune, and microbial factors. Rather than restating the pyroptotic machinery described in section 2.1.1, this section focuses on where that machinery is engaged in disease and on how strong the microbial evidence is. In IBD, pyroptotic activity localizes predominantly to IECs and lamina propria macrophages, where inflammasome activation, caspase-1 cleavage, GSDMD pore formation and IL-1β/IL-18 release accompany mucosal injury and immune dysregulation (Bao et al., 2024; Guo et al., 2025; Wang et al., 2021). Most of these human data are cross-sectional and therefore correspond to level 3–4 evidence, with microbial causality inferred rather than demonstrated. Notably, monocarboxylate transporter 4 (MCT4) has been implicated in promoting caspase-1-mediated pyroptosis via ERK1/2-NF-κB signaling, aggravating intestinal inflammation in IBD (Wang et al., 2021). An extraintestinal example is provided by polycystic ovary syndrome, in which gasdermin E-dependent macrophage pyroptosis associated with gut dysbiosis has been reported to disturb steroidogenesis and granulosa-cell survival (Huang et al., 2022); because microbiota transfer experiments were used, this represents level 2 evidence, although the downstream ovarian effects remain correlative.

**Figure 1:**
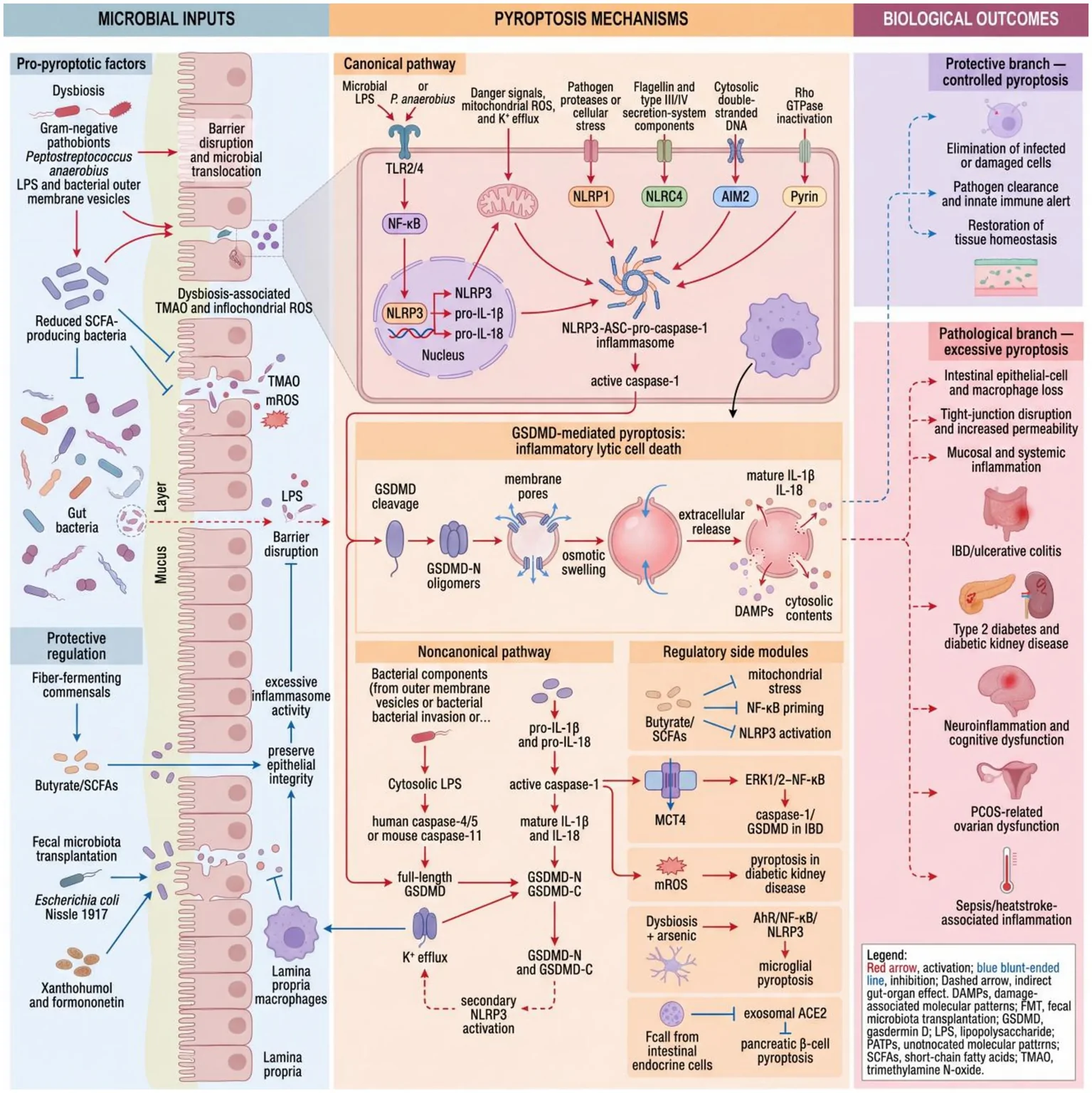
Gut microbiota regulation of pyroptosis in intestinal and systemic diseases. Gut dysbiosis, pathogen-associated molecular patterns, and microbial products provide inflammasome-priming and activating signals through canonical and noncanonical pathways. Canonical inflammasomes activate caspase-1, whereas cytosolic lipopolysaccharide activates human caspase-4/5 or murine caspase-11. Both pathways converge on GSDMD cleavage. GSDMD-N forms plasma-membrane pores, causing ionic imbalance, osmotic swelling, membrane rupture, and release of IL-1β, IL-18, DAMPs, and cytosolic contents, thereby defining pyroptosis as inflammatory lytic cell death. Controlled pyroptosis supports pathogen clearance, whereas excessive activation disrupts epithelial barriers and promotes intestinal and systemic inflammation. Microbiota-derived metabolites and microbiota-restoring interventions may suppress excessive inflammasome activity and preserve tissue homeostasis.

Beyond intestinal disorders, pyroptosis regulated by gut microbiota also plays a significant role in metabolic diseases such as type 2 diabetes (T2D) and its complications, including cognitive dysfunction. T2D is associated with gut microbiota dysbiosis, increased intestinal permeability, and chronic systemic inflammation, which contribute to metabolic derangements and organ damage (Yang et al., 2021). Pyroptosis of various cell types, including macrophages and IECs, is implicated in the inflammatory milieu of T2D. Emerging evidence indicates that gut microbiota-derived metabolites, such as trimethylamine N-oxide (TMAO), modulate pyroptosis pathways, influencing insulin resistance and neuroinflammation (Chen Z. et al., 2023; Zhang and Qin, 2026). For instance, chronic arsenic exposure induces cognitive impairment and Alzheimer’s disease-like pathology via gut microbiota-mediated microglial pyroptosis through the AhR/NF-κB/NLRP3 axis, with increased intestinal permeability facilitating neuroinflammation (Qu et al., 2026). Similarly, gut microbiota dysbiosis in diabetic kidney disease promotes pyroptosis via mitochondrial ROS (mROS)-NLRP3 inflammasome activation, which can be ameliorated by TCM formulations targeting gut-derived metabolites (Yi et al., 2023). Moreover, gut microbiota-derived metabolites such as xanthohumol and formononetin inhibit macrophage pyroptosis, reducing systemic inflammation in heatstroke and sepsis models, suggesting the therapeutic potential of modulating pyroptosis in metabolic and inflammatory conditions (Chen X. et al., 2023; Huang et al., 2026). The gut-brain axis is further exemplified by studies showing that small intestinal endocrine cell-derived exosomal ACE2 suppresses β-cell pyroptosis, improving islet function in metabolic stress (Yang et al., 2024). Collectively, these findings underscore the critical role of gut microbiota in regulating pyroptosis pathways that contribute to metabolic disease progression and associated cognitive dysfunction. Therapeutic strategies aimed at restoring gut microbial balance and inhibiting pyroptosis hold promise in managing metabolic diseases and their neurological complications (Figure 1).

## 3 Interaction between gut microbiota and ferroptosis

### 3.1 Mechanisms of ferroptosis and its pathological implications

Ferroptosis is a distinct form of regulated cell death characterized primarily by iron-dependent lipid peroxidation leading to membrane damage and cell demise. Unlike apoptosis or necrosis, ferroptosis is driven by the accumulation of lethal lipid peroxides on polyunsaturated fatty acid (PUFA)-containing phospholipids in cellular membranes, a process tightly regulated by iron metabolism and antioxidant defenses. Central to ferroptosis regulation are key molecules such as glutathione peroxidase 4 (GPX4) and acyl-CoA synthetase long-chain family member 4 (ACSL4). GPX4 acts as a pivotal antioxidant enzyme that reduces lipid hydroperoxides to non-toxic lipid alcohols, thereby preventing ferroptotic cell death. Its inactivation or depletion leads to unchecked lipid peroxidation and ferroptosis initiation. Conversely, ACSL4 facilitates the incorporation of PUFAs into membrane phospholipids, thus promoting susceptibility to lipid peroxidation and ferroptosis. The intricate balance between these regulators dictates cellular vulnerability to ferroptosis. Furthermore, oxidative stress interplays with ferroptosis by enhancing ROS generation and lipid peroxidation, which amplifies ferroptotic signaling. Iron overload exacerbates this process by catalyzing Fenton reactions that produce highly reactive hydroxyl radicals, intensifying lipid peroxidation and membrane damage. Ferroptosis can be triggered via extrinsic mechanisms, such as inhibition of the cystine/glutamate antiporter system Xc- or activation of iron transporters like transferrin, or intrinsic pathways involving direct suppression of intracellular antioxidants like GPX4. This multifaceted regulation underscores the complexity of ferroptosis as a cellular fate determinant (Hadian and Stockwell, 2020; Tang and Kroemer, 2020).

Pathologically, ferroptosis has been implicated in a broad spectrum of diseases, prominently including liver diseases, neurodegenerative disorders, and cardiovascular diseases (CVDs). In liver pathology, ferroptosis contributes to acute liver injury and chronic liver diseases by mediating hepatocyte death through iron overload and oxidative stress. Experimental models using liver-specific Fbxl5-null mice have demonstrated that iron accumulation induces severe liver damage via ferroptosis, characterized by elevated liver enzymes, histopathological alterations, and lipid peroxidation. Transcriptomic analyses have identified hepatic gene signatures associated with ferroptosis, linking ferroptotic pathways to liver injury progression and suggesting potential therapeutic targets (Fan et al., 2022; Matsumoto et al., 2025). In neurodegenerative diseases such as Parkinson’s and Alzheimer’s diseases, ferroptosis is increasingly recognized as a critical contributor to neuronal loss. The excessive iron accumulation and lipid peroxidation observed in affected brain regions promote ferroptotic death of neurons. For instance, in Parkinson’s disease models, nicotinamide adenine dinucleotide phosphate (NADPH) oxidase 4 (NOX4) exacerbates ferroptosis and neuroinflammation, leading to dopaminergic neuron death. Targeting ferroptosis regulators like GPX4 and ferroptosis suppressor protein 1 (FSP1) offers promising neuroprotective strategies (Chen and Xie, 2020; Lin et al., 2025; Zhang J. B. et al., 2023). Similarly, in CVDs, ferroptosis contributes to myocardial injury, ischemia–reperfusion damage, and pathological remodeling. Excessive β-adrenergic stimulation induces ferroptosis in cardiomyocytes via glutathione depletion and GPX4 degradation, accompanied by increased heme oxygenase 1 (HO-1) expression and labile iron accumulation. Inhibiting ferroptosis with agents like ferrostatin-1 or targeting upstream regulators mitigates cardiac necrosis and heart failure, highlighting ferroptosis as a novel therapeutic target in cardiology (Chen Y. et al., 2023; Zhang L. L. et al., 2022; Zhou X. et al., 2025). Collectively, these findings emphasize that ferroptosis, through its iron-dependent lipid peroxidation and oxidative stress mechanisms, plays a central role in the pathogenesis of diverse organ-specific diseases, opening avenues for targeted interventions aimed at modulating ferroptotic pathways for therapeutic benefit.

### 3.2 Gut microbiota regulates ferroptosis via metabolites

The gut microbiota exerts a profound influence on ferroptosis, primarily through its metabolic products, which modulate iron metabolism, oxidative stress, and lipid peroxidation pathways critical to ferroptotic cell death. Alterations in gut microbial composition can disrupt systemic iron homeostasis, thereby impacting ferroptosis induction. For instance, dysbiosis often leads to iron metabolism disorders that facilitate iron accumulation, a hallmark of ferroptosis, thus promoting its initiation. This is supported by evidence showing that pathogenic bacteria tend to enhance ferroptosis by increasing iron availability and oxidative stress, whereas beneficial microbes often exert protective effects by maintaining iron balance and antioxidant defenses (Yao and Li, 2023).

Gut microbiota also regulates the redox state of host tissues, influencing the expression and activity of GPX4, a key enzyme that detoxifies lipid peroxides and suppresses ferroptosis. Certain microbial metabolites, including SCFAs like butyrate and valerate, have been shown to enhance GPX4 expression, thereby reducing ferroptotic sensitivity. For example, butyric acid and valeric acid mitigate neuroinflammation and neuronal ferroptosis in stress models by modulating inflammatory signaling pathways and promoting antioxidant defenses, including GPX4 upregulation (Ma X. et al., 2025; Wang Z. et al., 2025). Similarly, the gut microbiota-derived metabolite capsiate activates transient receptor potential cation channel subfamily V member 1 (TRPV1), which enhances GPX4 expression and inhibits ferroptosis in intestinal ischemia/reperfusion injury models (Deng et al., 2021). These findings highlight the pivotal role of microbial metabolites in maintaining redox homeostasis and ferroptosis resistance through GPX4 modulation.

Furthermore, gut microbiota influences ferroptosis by regulating the host’s oxidative stress levels and lipid metabolism. Dysbiosis can lead to increased production of ROS and lipid peroxides, exacerbating ferroptotic cell death. Conversely, certain probiotics and their metabolites restore antioxidant capacity and reduce lipid peroxidation, thus protecting against ferroptosis-related tissue injury (Hu et al., 2024; Wang X. et al., 2025). For instance, therapeutic agents like baicalin improve gut microbial balance and lipid metabolism, promoting GPX4 and SLC7A11 expression while suppressing ACSL4 and ROS accumulation, effectively inhibiting ferroptosis in neuronal cells (Ren et al., 2025). Additionally, microbial metabolites can regulate iron transport proteins such as ferroportin (FPN1) and transferrin receptor 1 (TFR1), further modulating iron availability and ferroptosis susceptibility (Liu et al., 2026; Tian et al., 2026). The molecular basis of ferroptosis itself is not restated here; it is described in section 2.2.1, and this section is confined to the microbial and metabolite layer that sets the ferroptotic threshold. It should also be noted that baicalin and comparable compounds inhibit lipid peroxidation directly in cell-free and germ-free systems, so the accompanying microbial shifts constitute level 3 rather than level 1 evidence unless the effect is lost upon microbiota depletion. Because most microbiota–ferroptosis evidence derives from *in vitro* systems or animal models, its clinical relevance should be interpreted cautiously and validated in human cohorts using integrated microbiome, metabolomic, and tissue-based ferroptosis readouts.

In summary, gut microbiota-derived metabolites regulate ferroptosis through both direct metabolic signaling and indirect inflammatory-metabolic remodeling. Direct regulation occurs when microbial metabolites engage defined host receptors or metabolic enzymes, such as SCFA-mediated GPR43/GPR109A signaling or HDAC inhibition, bile acid signaling through TGR5/FXR/S1PR2-dependent pathways, and tryptophan metabolite–AhR signaling. Indirect regulation occurs when dysbiosis first alters epithelial permeability, bile acid pools, mitochondrial function, systemic iron distribution, or immune-cell polarization, thereby secondarily changing the ferroptotic threshold through GPX4, SLC7A11, ACSL4, TFR1, or NRF2-related pathways. This distinction is important because microbial metabolites are not uniformly protective or pathogenic. These apparently conflicting observations suggest that the effects of microbial metabolites depend on metabolite species, dose, receptor usage, inflammatory priming, target cell type, diet, animal strain, and microbiota background. Therefore, future studies should distinguish metabolite-driven primary regulation of RCD machinery from secondary changes caused by inflammatory or metabolic remodeling. The regulatory roles of gut microbiota and microbial metabolites in ferroptosis, including their molecular targets and disease implications, are summarized in Table 2.

### 3.3 Ferroptosis and diseases associated with gut microecological imbalance

Reflux esophagitis (RE) is a common inflammatory condition of the esophagus caused by the retrograde flow of gastric contents, leading to mucosal damage. Emerging evidence implicates ferroptosis, an iron-dependent form of RCD characterized by lipid peroxidation, in the pathogenesis of RE. Proteomic analysis of reflux esophagitis has demonstrated ACSL4 activation and ferroptosis-associated esophageal mucosal injury, which currently provides the most direct evidence in this setting (Liu et al., 2022). Beyond this observation, direct studies of ferroptosis in RE remain scarce, and no study has yet tested whether the esophageal or gastric microbiota is required for ferroptotic mucosal injury; the mechanistic account that follows is therefore extrapolated from other gastrointestinal inflammatory conditions and should be read as a hypothesis (level 4 evidence). Insights from related gastrointestinal inflammatory diseases suggest that ferroptosis contributes to mucosal injury through oxidative stress and disruption of epithelial barrier integrity. The gut microbiota, a key regulator of intestinal and systemic homeostasis, may influence ferroptosis by modulating iron metabolism, ROS generation, and the inflammatory milieu. Dysbiosis, or microbial imbalance, can exacerbate oxidative stress and lipid peroxidation, thereby potentially favoring ferroptotic cell death in the esophageal epithelium. For instance, certain bacterial taxa produce metabolites that either enhance antioxidant defenses or generate pro-oxidant molecules, affecting the susceptibility of epithelial cells to ferroptosis. Therapeutic modulation of the microbiota, through probiotics or dietary interventions, may restore microbial balance and attenuate ferroptosis-mediated mucosal damage in RE. Targeting the microbiota–ferroptosis axis is therefore a plausible but as yet unvalidated strategy in RE, and further mechanistic studies are warranted to delineate specific microbial species and metabolites involved in ferroptosis regulation within the esophageal microenvironment.

**Figure 2:**
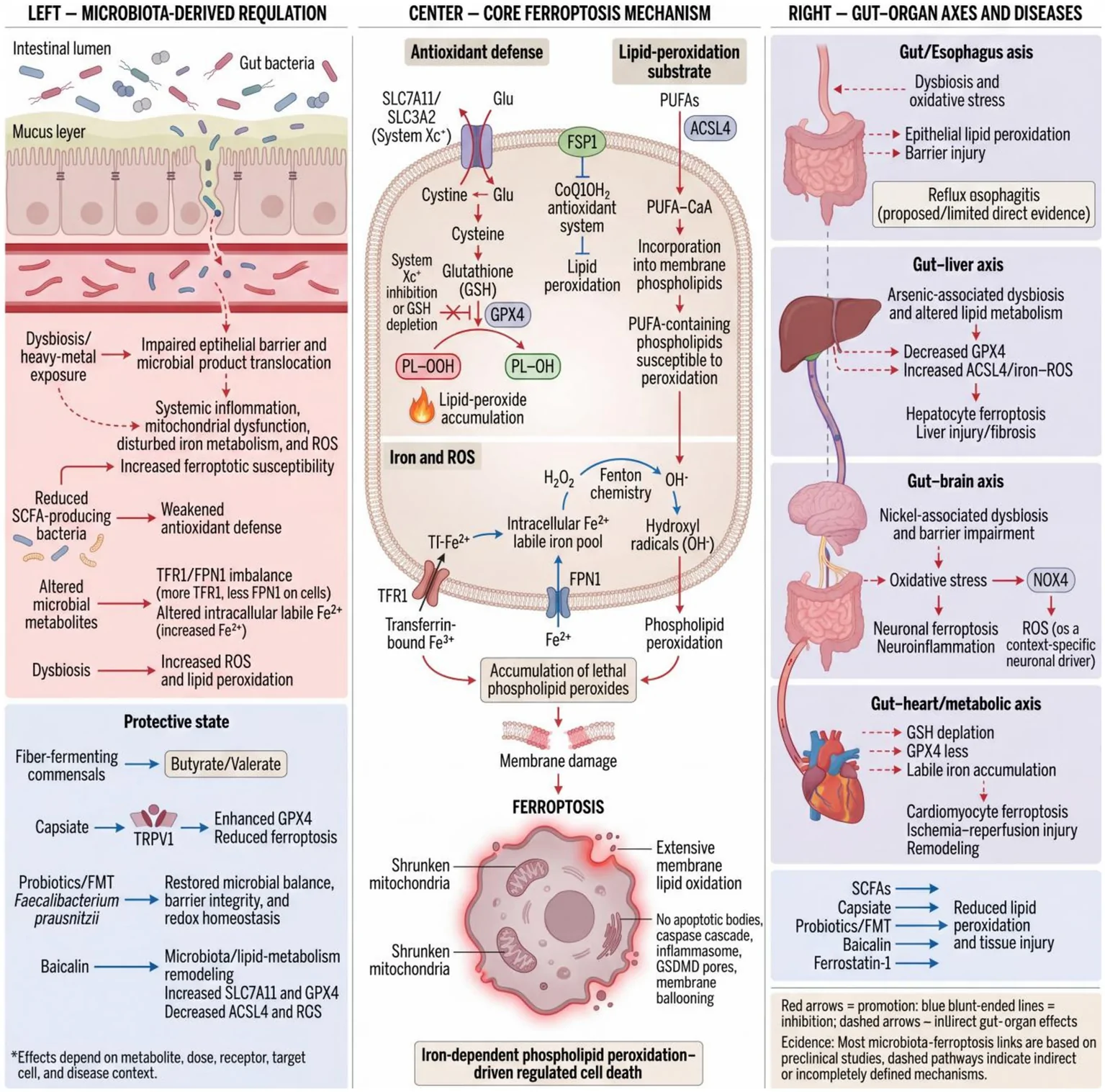
Gut microbiota–ferroptosis interactions in local and systemic diseases. Gut dysbiosis and microbial metabolites influence ferroptotic susceptibility by altering epithelial permeability, systemic iron handling, mitochondrial oxidative stress, antioxidant capacity, and lipid metabolism. Ferroptosis results from the convergence of an increased labile iron pool, ACSL4-dependent enrichment of PUFA-containing membrane phospholipids, and inadequate antioxidant defense through the system Xc^−^–GSH–GPX4 and FSP1–CoQ10H2 pathways. The resulting accumulation of lethal phospholipid peroxides causes membrane damage and ferroptotic cell death. SCFAs, capsiate, probiotics, FMT, and selected natural products may reinforce antioxidant defenses, whereas dysbiosis and toxic exposures may promote ferroptosis in the gastrointestinal tract, liver, brain, and cardiovascular system. Many microbiota–ferroptosis relationships remain preclinical or context-dependent.

Heavy metal toxicity, particularly from metals such as nickel, arsenic, and bromoacetic acid, is a significant contributor to liver injury and dysfunction. Recent studies have elucidated that ferroptosis plays a pivotal role in mediating hepatocellular damage induced by heavy metal exposure. For example, nickel sulfate (NiSO4) exposure in murine models leads to neurotoxicity accompanied by ferroptosis, gut microbiota dysbiosis, and compromised intestinal barrier integrity, highlighting a gut-brain axis involvement (Shen et al., 2025a). Similarly, arsenic exposure disrupts hepatic lipid metabolism and induces ferroptosis, with concurrent alterations in gut microbiota composition and metabolite profiles, suggesting a gut-liver axis in mediating toxicity (Shao et al., 2024). Mechanistically, heavy metals promote iron accumulation and lipid peroxidation in hepatocytes, triggering ferroptosis through downregulation of GPX4 and upregulation of ACSL4. The gut microbiota modulates this process by influencing systemic iron homeostasis, oxidative stress responses, and inflammatory signaling. Dysbiosis induced by heavy metals can exacerbate intestinal permeability, allowing translocation of microbial products that further aggravate hepatic inflammation and ferroptosis. Therapeutic interventions targeting the gut microbiota, such as FMT or administration of beneficial bacteria like *Faecalibacterium prausnitzii*, have demonstrated protective effects by suppressing ferroptosis and restoring metabolic balance in liver injury models (Zhang Y. et al., 2025). Moreover, natural compounds such as total *Sanghuangporus vaninii* extract ameliorate liver fibrosis by inhibiting hepatocyte ferroptosis and remodeling gut microbiota homeostasis (Gao et al., 2025). These findings underscore the integral role of the gut microbiota in modulating heavy metal-induced hepatic ferroptosis and suggest that microbiota-targeted therapies may offer promising strategies to mitigate liver toxicity. Future research should focus on identifying specific microbial taxa and metabolites that regulate ferroptotic pathways in the context of heavy metal exposure, as well as elucidating the molecular mechanisms underlying gut-liver crosstalk in ferroptosis regulation (Figure 2).

## 4 Other RCD mechanisms and their association with the gut microbiota

### 4.1 Crosstalk regulation among pyroptosis, necroptosis, and autophagy

**Figure 3:**
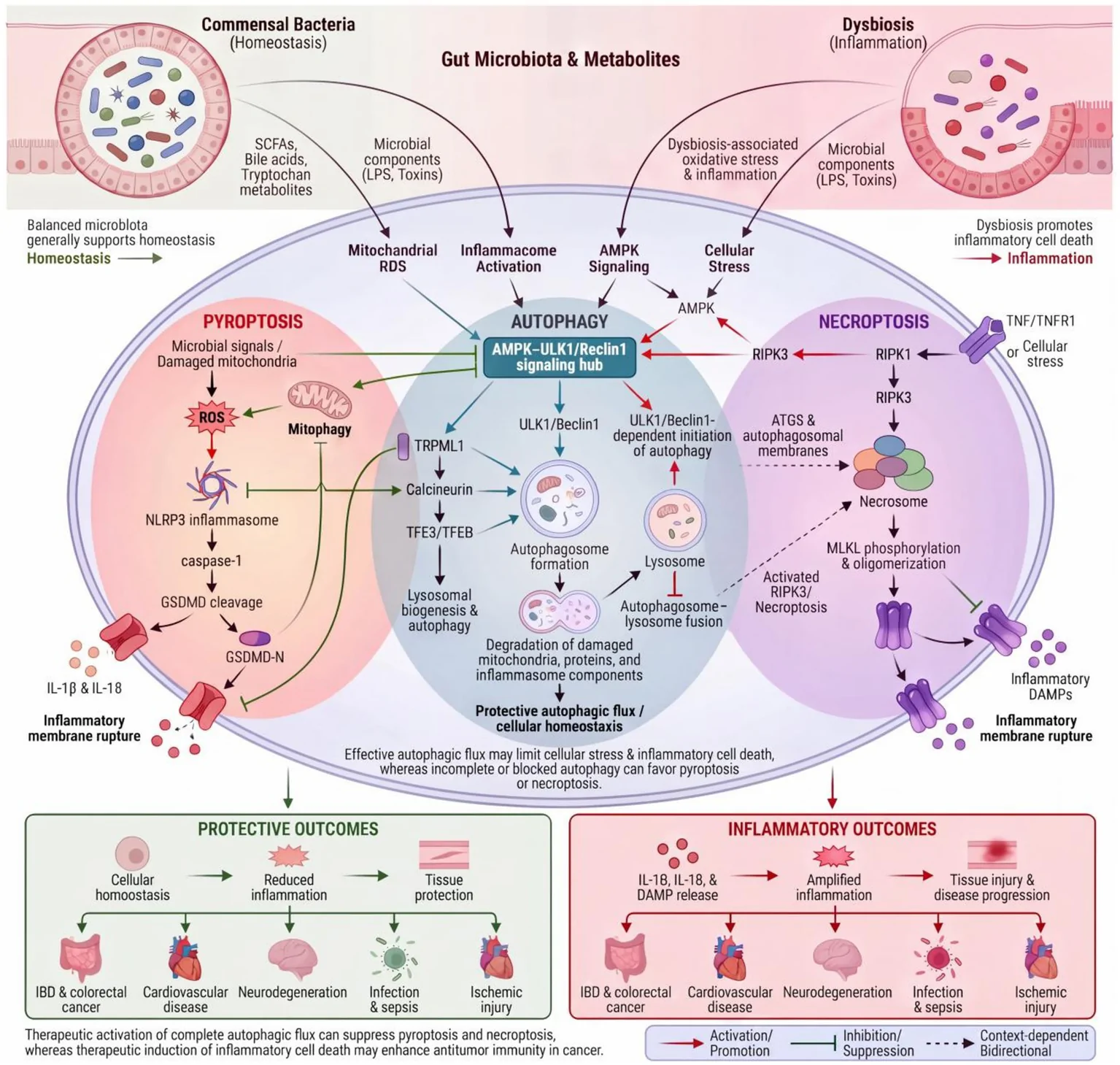
Gut microbiota-regulated crosstalk among pyroptosis, necroptosis, and autophagy. Gut microbial components, metabolites, and dysbiosis-associated signals regulate mitochondrial dysfunction, oxidative stress, inflammasome activation, and AMPK signaling. Autophagy and mitophagy generally suppress pyroptosis by removing damaged mitochondria, reducing ROS generation, and degrading inflammasome components, thereby limiting NLRP3 activation, GSDMD cleavage, and IL-1β/IL-18 release. Autophagy interacts bidirectionally with necroptosis: Autophagy-related proteins and autophagosomal membranes can facilitate RIPK1–RIPK3 necrosome assembly, whereas RIPK3–AMPK signaling promotes ULK1/Beclin1-dependent autophagy initiation. Conversely, necroptosis may impair autophagic flux by disrupting autophagosome–lysosome fusion. The balance among these pathways determines cellular homeostasis, inflammatory responses, tissue injury, disease progression, and antitumor immunity. AMPK, AMP-activated protein kinase; DAMPs, damage-associated molecular patterns; GSDMD, gasdermin D; MLKL, mixed lineage kinase domain-like pseudokinase; NLRP3, NLR family pyrin domain-containing 3; RIPK, receptor-interacting protein kinase; ROS, reactive oxygen species; TFEB, transcription factor EB; TFE3, transcription factor E3; ULK1, Unc-51-like autophagy-activating kinase 1.

RCD encompasses a variety of molecularly distinct mechanisms, including pyroptosis, necroptosis, and autophagy, each with unique roles in cellular homeostasis and disease pathogenesis. Pyroptosis is a proinflammatory form of RCD characterized by GSDM-mediated plasma membrane pore formation, leading to cell swelling, lysis, and release of inflammatory cytokines such as IL-1β and IL-18. Necroptosis, another regulated necrotic cell death modality, is orchestrated by receptor-interacting protein kinases RIPK1 and RIPK3 and the pseudokinase MLKL, culminating in membrane rupture and inflammation. Autophagy, in contrast, is primarily a cytoprotective catabolic process that degrades damaged organelles and proteins via lysosomal pathways, thereby maintaining cellular homeostasis under stress. It should be emphasized that autophagy is, in most contexts, a homeostatic and cytoprotective degradative process rather than a cell-death modality in its own right. The designation autophagy-dependent cell death is reserved for the narrow situations in which death is mechanistically executed by the autophagic machinery and is prevented by knockdown of core ATG genes rather than merely delayed (Galluzzi et al., 2018). In essentially all of the microbiota-related studies discussed below, autophagy acts as an upstream regulatory process that sets the threshold for pyroptosis or necroptosis, and it is referred to in that sense throughout this section. Emerging evidence reveals intricate crosstalk among these pathways, where autophagy can modulate pyroptosis and necroptosis, and vice versa, influencing disease outcomes (Figure 3).

Molecularly, autophagy intersects with pyroptosis and necroptosis at multiple levels. For instance, autophagy can negatively regulate pyroptosis by degrading inflammasome components or damaged mitochondria, thereby reducing ROS and inflammasome activation. Studies have demonstrated that autophagy induction suppresses NLRP3 inflammasome activation and GSDMD cleavage, attenuating pyroptotic cell death in various contexts including sepsis, liver disorders, and CVDs (Guo et al., 2021; He et al., 2021; Shkarina et al., 2022). Conversely, inhibition of autophagy exacerbates pyroptosis, highlighting its protective role. Similarly, autophagy modulates necroptosis by regulating the stability and activation of key necroptotic proteins. For example, autophagy-related proteins such as ATG5 facilitate necrosome formation, and autophagosomes serve as platforms for RIPK1/RIPK3 interactions, promoting necroptosis under certain conditions (Lee et al., 2022; Sun et al., 2022). Intriguingly, necroptosis can also impair autophagic flux, as evidenced by RIP3-mediated disruption of autophagosome-lysosome fusion, indicating bidirectional regulation (Liu et al., 2023a).

The AMP-activated protein kinase (AMPK) pathway emerges as a central signaling hub linking these processes. RIPK3 has been identified as a kinase activating AMPK, which in turn phosphorylates autophagy-initiating kinases ULK1 and Beclin1, initiating autophagy (Wu et al., 2021). However, during TNF-induced necroptosis, autophagic flux is blocked at the lysosomal degradation stage, suggesting that necroptosis can initiate early autophagy events but inhibit their completion, thereby influencing cell fate decisions. Transcription factors such as TFE3 and TFEB also mediate the crosstalk by regulating autophagy and oxidative stress responses, which indirectly influence pyroptosis and necroptosis (Lou et al., 2022; Xu et al., 2021). For instance, cyclic helix B peptide (CHBP) promotes autophagy via AMPK-TRPML1-calcineurin-TFE3 signaling, suppressing pyroptosis and necroptosis in ischemic injury models (Lou et al., 2022).

Gut microbiota and their metabolites further modulate these cell death pathways in the intestinal environment. Dysbiosis can influence the balance between autophagy and proinflammatory cell death, impacting diseases such as IBD and colorectal cancer (Zhou et al., 2023). Moreover, non-coding RNAs have been implicated in regulating the transcriptional and post-transcriptional control of autophagy, pyroptosis, and necroptosis, thereby affecting tumorigenesis and metastasis (Han et al., 2024; Liu et al., 2021).

In pathological contexts such as neurodegenerative diseases, CVDs, and infections, the interplay among pyroptosis, necroptosis, and autophagy determines the extent of inflammation and tissue damage. For example, in spinal cord injury, therapeutic agents that enhance autophagy concurrently suppress pyroptosis and necroptosis, improving functional outcomes (Gao et al., 2022; Xu et al., 2023; Zhang H. et al., 2023). Similarly, in Parkinson’s disease models, activation of autophagy inhibits neuroinflammation mediated by pyroptosis and necroptosis (Zhang M. et al., 2023). In cancer, the induction of necroptosis and pyroptosis, often in conjunction with autophagy modulation, represents a promising strategy to overcome apoptosis resistance and enhance antitumor immunity (Tang et al., 2020; Woo et al., 2020).

### 4.2 Emerging forms of cell death: cuproptosis, disulfidptosis, and others

The recent identification of novel RCD modalities such as cuproptosis and disulfidptosis has expanded our understanding of cellular demise beyond classical apoptosis, pyroptosis, and ferroptosis. Cuproptosis is a copper-dependent form of RCD in which copper binds directly to lipoylated components of the tricarboxylic acid (TCA) cycle, causing aggregation of these proteins, loss of iron–sulfur cluster proteins, proteotoxic stress and cell death; ferredoxin 1 (FDX1) is a pivotal upstream regulator (Tsvetkov et al., 2022; Chen et al., 2022; Cobine and Brady, 2022; Duan and He, 2022; Meena et al., 2025; Wang D. et al., 2023). The remainder of this section should be read as an emerging conceptual framework rather than as established mechanism. To our knowledge, no study has yet demonstrated microbiota-induced cuproptosis or disulfidptosis *in vivo*, and no work has shown that either modality is lost upon microbiota depletion. The connections proposed below are hypotheses generated by juxtaposing two separate literatures—one on microbial metal and amino-acid metabolism, the other on copper- and disulfide-dependent death—and correspond to level 4 evidence throughout.

Gut microbiota might plausibly influence cuproptotic susceptibility through three interconnected layers, none of which has yet been tested against a cuproptotic endpoint. First, the gut microbiota participates in host copper handling: dietary copper reshapes microbial diversity and correlates with inflammatory cytokines, while antibiotic-mediated microbiota depletion alters intestinal copper transporter expression and copper isotopic composition (Miller et al., 2019; Zhang et al., 2017). Second, excessive luminal copper damages barrier function and alters microbiota-derived metabolites, which could amplify mucosal inflammation and increase the exposure of epithelial and immune cells to copper-associated stress (Liao et al., 2021). Third, because cuproptosis requires active mitochondrial respiration and lipoylated TCA-cycle proteins, microbial metabolites that modify mitochondrial substrate flux, bile-acid signaling, SCFA availability or cytokine tone could in principle determine whether copper accumulation is detoxified or becomes lethal. A possible interface with pyroptosis and ferroptosis follows from the same logic: copper has been reported to regulate canonical NLRP3 inflammasome activation (Deigendesch et al., 2018), and copper-induced loss of iron–sulfur cluster proteins together with disturbed glutathione-dependent redox buffering could increase lipid-peroxidation vulnerability. Each of these steps is currently inferential.

Disulfidptosis is mechanistically centered on SLC7A11-dependent cystine uptake and redox imbalance. Under nutrient-replete conditions, SLC7A11 imports cystine to support cysteine and glutathione synthesis, thereby protecting cells from ferroptosis. However, in SLC7A11-high cells under glucose starvation, excessive cystine import becomes detrimental because the reduction of cystine to cysteine consumes NADPH. When glucose-derived pentose phosphate pathway activity is insufficient to regenerate NADPH, cystine and other disulfide molecules accumulate, inducing abnormal disulfide bonding in actin cytoskeletal proteins, F-actin network collapse and a distinct form of cell death termed disulfidptosis (Liu et al., 2023b). SLC7A11 expression level dictates whether cystine uptake functions as an antioxidant program or becomes a pro-death liability under NADPH-depleting stress (Yan et al., 2023).

A link between the gut microbiota and disulfidptosis has not been demonstrated experimentally, but is conceivable through amino-acid and glutathione metabolism. The gut microbiota modulates host amino-acid and glutathione metabolism in multiple tissues, providing a plausible route by which dysbiosis could alter cysteine/cystine availability and cellular reducing capacity (Mardinoglu et al., 2015). In inflamed mucosa or tumors, microbial dysbiosis may simultaneously reshape glucose utilization, cysteine supply, glutathione turnover and NADPH-generating pathways; if such remodeling increased cystine dependence while limiting NADPH regeneration, SLC7A11-high epithelial or tumor cells could in principle shift from ferroptosis resistance toward disulfidptosis susceptibility. SLC7A11 would then act as a mechanistic switch between the two modalities. This model is testable—for example by combining gnotobiotic colonization with SLC7A11 manipulation and F-actin disulfide readouts—but remains untested.

Pathologically, cuproptosis and disulfidptosis are best regarded as emerging, context-dependent extensions of the microbiota-regulated cell-death network rather than as established mechanisms. In intestinal inflammation and colitis-associated cancer, copper metabolism has been linked to inflammatory regulation and tumorigenesis; COMMD1, a copper metabolism-related regulator, restrains NF-κB-driven inflammatory genes and protects against colitis and colitis-associated cancer (Li et al., 2014). This supports a conceptual link among copper handling, mucosal inflammation and cell-death susceptibility, but does not demonstrate microbiota-induced cuproptosis. Similarly, disulfidptosis may become relevant in colorectal and other metabolically stressed tumors where hypoxia, nutrient deprivation, high SLC7A11 expression and microbial metabolic remodeling coexist. Defining whether these modalities are genuinely microbiota-regulated is a priority for future work and is listed among the research directions in section 2.5.4.

### 4.3 Interaction between immune cell polarization and RCD

The intricate interplay between immune-cell polarization and RCD is a key mechanism by which gut microbiota shapes inflammatory and antitumor responses. In this process, macrophage polarization acts as an intermediate amplifier rather than an isolated immune phenotype. Dysbiosis-associated PAMPs and inflammatory cytokines can promote TLR–NF-κB- and STAT1/IRF-dependent inflammasome priming, favoring M1-like polarization and increasing pyroptotic susceptibility. Conversely, microbial metabolites such as butyrate, secondary bile acids, and tryptophan-derived AhR ligands may support tolerogenic or tissue-repair programs through GPCR signaling, HDAC inhibition, mitochondrial metabolic rewiring, and IL-10-associated pathways. Gut microbes regulate macrophage polarization by shaping the local immune microenvironment, thereby affecting the balance between pro-inflammatory M1 and anti-inflammatory M2 macrophages, which in turn modulate pyroptotic and ferroptotic pathways. For instance, dysbiosis-induced metabolites and inflammatory signals can promote M1 polarization, which is associated with enhanced pyroptosis and ferroptosis, leading to tissue damage and chronic inflammation. Conversely, signals favoring M2 polarization tend to suppress these cell death pathways, facilitating tissue repair and immune homeostasis.

The PD-1/PD-L1 axis further connects microbial immune remodeling with RCD outcomes. It should be emphasized that PD-1/PD-L1 is an immune-checkpoint signaling axis rather than a cell-death mechanism in its own right; it is discussed here only because checkpoint signaling shapes the immune context in which RCD occurs. Beyond T-cell exhaustion, PD-1 expression on tumor-associated macrophages has been shown to restrain phagocytosis, whereas PD-1/PD-L1 blockade restores macrophage antitumor activity (Gordon et al., 2017). Therefore, dysbiosis-driven cytokine production, epithelial PD-L1 expression, antigen presentation, and macrophage polarization may jointly determine whether RCD promotes inflammatory damage, immune tolerance, or antitumor immunity (Li S. et al., 2025; Zuo and Wan, 2022). Moreover, microbial components such as *Lactobacillus rhamnosus* DNA can induce PD-L1 expression in IECs, promoting apoptosis of activated Th2 cells and modulating immune polarization (Song et al., 2025). A recent clinical and mechanistic study in esophageal squamous cell carcinoma shows how a single microbial metabolite can determine checkpoint-blockade outcome through a defined host receptor. Across 122 patients receiving neoadjuvant anti-PD-1 therapy, Ligilactobacillus salivarius was enriched in non-responders, and its tryptophan-derived metabolite indole-3-lactic acid (ILA) activated AhR and downregulated NF-κB signaling in tumor-infiltrating NKG7 + CD8 + precursor-exhausted T cells, driving terminal exhaustion and impairing antitumor immunity; isogenic ILA-deficient strains abolished both ILA production and immune resistance, and pharmacological NF-κB activation restored T-cell function and reversed resistance (Zhou et al., 2026). This positions AhR engagement by tryptophan-derived metabolites as context-dependent rather than uniformly protective, since the same receptor axis preserves intraepithelial lymphocyte viability in the mucosa (Liu R. et al., 2024) yet suppresses effector CD8 + T cells within the tumor microenvironment. In pathological conditions like liver transplant rejection, TDO2 deficiency shifts macrophage polarization toward the M1 phenotype and elevates PD-1/PD-L1 expression, exacerbating immune responses and RCD (Li S. et al., 2025). The crosstalk extends to cancer, where M1-polarized macrophage-derived exosomes carrying miR-142-3p suppress tumor growth and immune escape by regulating the HMGB1-mediated PD-1/PD-L1 checkpoint, linking macrophage polarization with pyroptosis and immune modulation (Wei et al., 2025). Furthermore, the gut microbiota influences these dynamics by modulating metabolic and inflammatory pathways that trigger pyroptosis and ferroptosis, which reciprocally affect macrophage polarization and immune cell death, creating a feedback loop that shapes disease progression (Zhang and Qin, 2026).

Collectively, these findings underscore a complex network whereby gut microbiota-mediated signals regulate macrophage polarization, which in turn modulates RCD pathways such as pyroptosis and ferroptosis, influencing immune responses, inflammation, and disease outcomes. Therapeutic strategies targeting this axis, including modulation of PD-1/PD-L1 signaling, metabolic pathways, and microbial composition, hold promise for treating inflammatory and neoplastic diseases linked to dysregulated immune cell death and polarization.

## 5 Disease models and clinical correlations of gut microbiota regulating RCD

Although impaired epithelial barrier function and microbial product leakage represent shared upstream events, different organs translate these microbial signals into distinct RCD outcomes. In the gut–liver axis, portal circulation exposes hepatocytes and Kupffer cells to microbial metabolites, bile acids, and TMAO, linking dysbiosis to mitochondrial stress, inflammasome activation, and hepatocyte ferroptosis. In the gut–lung axis, circulating microbial products and immune mediators mainly affect alveolar macrophages, neutrophils, and epithelial–endothelial barrier injury, making macrophage pyroptosis and epithelial oxidative injury more prominent. In the gut–brain axis, microbial metabolites and systemic inflammation influence microglial activation and neuronal redox vulnerability, whereas in the gut–cardiovascular axis, TMAO and related metabolites primarily act on endothelial cells, plaque macrophages, vascular smooth muscle cells, and cardiomyocytes. Thus, intestinal barrier dysfunction should be regarded as a common entry point, while organ-specific target cells, vascular routes, dominant microbial metabolites, and local metabolic states determine the final RCD phenotype (Koeth et al., 2013).

### 5.1 Microbiota-cell death axis in metabolic diseases

In T2D, microbiota-regulated cell death involves distinct target cells in different complications rather than a single systemic pathway. In pancreatic and renal complications, mitochondrial ROS and NLRP3 activation increase the susceptibility of β cells, renal tubular cells, and infiltrating macrophages to pyroptosis. For example, TMAO, a gut microbiota-derived metabolite elevated in diabetic patients, exacerbates endothelial dysfunction and promotes pyroptosis and oxidative stress, contributing to vascular complications and neuroinflammation that underlie cognitive decline (Liu et al., 2025a; Yi et al., 2023). Notably, interventions targeting the gut microbiota-pyroptosis/ferroptosis axis, such as the use of Zuogui-Jiangtang-Yishen decoction, have shown promise in preventing diabetic kidney disease by suppressing TMAO-induced pyroptosis via the mROS-NLRP3 pathway (Yi et al., 2023). Collectively, these findings underscore a complex, bidirectional interplay where gut microbiota dysbiosis induces pyroptosis and ferroptosis, contributing to metabolic dysfunction and neurodegeneration in diabetes, while therapeutic modulation of the microbiota offers a novel avenue for intervention. Future research integrating multi-omics and advanced models is essential to unravel precise molecular mechanisms and optimize microbiota-targeted therapies in diabetes-associated cognitive dysfunction (Zhang et al., 2024; Zhang and Qin, 2026).

Obesity and metabolic syndrome are characterized by chronic low-grade inflammation and metabolic disturbances, in which gut microbiota-mediated regulation of RCD pathways, particularly pyroptosis and ferroptosis, plays a critical role. Gut dysbiosis in obesity leads to increased intestinal permeability and elevated circulating endotoxins. This inflammatory cell death exacerbates insulin resistance and adipose tissue dysfunction, perpetuating metabolic syndrome (Kong et al., 2022; Zhang and Qin, 2026). Additionally, ferroptosis contributes to adipocyte and muscle cell dysfunction in obesity by promoting iron-dependent lipid peroxidation and oxidative stress. Experimental evidence demonstrates that interventions such as supplementation with *Akkermansia muciniphila* enhance the therapeutic efficacy of GLP-1 receptor agonists in metabolic dysfunction-associated steatotic liver disease (MASLD; formerly non-alcoholic fatty liver disease, NAFLD) by remodeling the gut microbiota and suppressing intestinal pyroptosis, highlighting the microbiota’s role in regulating cell death and metabolic inflammation (Gao et al., 2026). Furthermore, ferroptosis inhibitors like phloretin have shown efficacy in ameliorating oxidative stress and ferroptosis in adipose and muscle cells via the AMPK-PPAR signaling pathway, independent of gut microbiota modulation, suggesting multifaceted therapeutic strategies (Zhang et al., 2026). The gut microbiota-ferroptosis axis is also implicated in osteoarthritis progression linked to obesity, where dysbiosis promotes ferroptosis of chondrocytes, exacerbating joint degeneration (Wang T. et al., 2025). Moreover, iron overload, frequently observed in metabolic syndrome, exacerbates colitis and metabolic inflammation by inducing ferroptosis and altering gut microbiota composition, further linking microbial regulation of ferroptosis to metabolic disease pathogenesis (Gu et al., 2023). Collectively, these insights reveal that gut microbiota dysbiosis orchestrates metabolic syndrome progression by modulating pyroptosis and ferroptosis, which drive inflammation and tissue dysfunction. Therapeutic modulation of the microbiota and cell death pathways holds promise for managing obesity-related metabolic disorders, with ongoing research needed to elucidate precise molecular interactions and optimize clinical interventions (Kong et al., 2022; Yao and Li, 2023; Zhang and Qin, 2026).

### 5.2 CVDs and gut microbiota-regulated RCD

In cardiovascular disease, microbiota-regulated cell death is distinguished by the direct exposure of vascular and cardiac cells to circulating microbial metabolites, particularly trimethylamine N-oxide (TMAO). Among the gut microbiota-derived metabolites, TMAO has emerged as a pivotal molecule linking intestinal microbial metabolism with cardiovascular pathophysiology. TMAO is generated in the liver from trimethylamine (TMA), itself produced by gut microbes metabolizing dietary nutrients such as choline, phosphatidylcholine, and L-carnitine; the microbial dependence of this step is well established, since TMA and TMAO production is abolished by antibiotic suppression of the microbiota and restored after its recovery (Koeth et al., 2013; Tang et al., 2013), which places TMAO generation itself at level 2 evidence. Elevated circulating TMAO levels have been consistently associated with increased risk of atherosclerosis, myocardial infarction, heart failure, and arrhythmias (Tang et al., 2013; Liu et al., 2025a; Zhou et al., 2020). These clinical data are, however, observational, and residual confounding by renal function, diet and comorbidity has not been fully excluded; the link between circulating TMAO and cardiovascular cell death in humans therefore remains at level 4.

Mechanistically, TMAO has been reported to induce endothelial dysfunction, promote vascular inflammation, and enhance oxidative stress, thereby contributing to the progression of cardiovascular disorders. In cultured vascular cells and in rodent models, TMAO exposure also engages pyroptotic and ferroptotic pathways. TMAO-associated inflammasome activation in endothelial cells and macrophages has been linked to IL-1β/IL-18 release, vascular inflammation and plaque instability in atherosclerosis, while TMAO-associated disruption of redox and iron homeostasis has been linked to cardiomyocyte and vascular ferroptosis in myocardial ischemia–reperfusion injury and heart failure. The underlying molecular machinery is described in sections 2.1.1 and 2.2.1 and is not restated here. Importantly, most of these studies administered TMAO exogenously rather than manipulating the microbiota, so they establish that TMAO is sufficient to lower the cell-death threshold but not that endogenous microbial TMAO production is necessary for cardiovascular cell death *in vivo*.

The relevant cell-death outcome varies according to cardiovascular compartment. In the vascular endothelium, TMAO-associated inflammasome activation and pyroptosis impair barrier integrity and facilitate leukocyte recruitment. Within atherosclerotic plaques, macrophage pyroptosis contributes to necrotic-core expansion and plaque instability, whereas ferroptotic injury in endothelial and vascular smooth-muscle cells promotes oxidative damage and vascular calcification. In the myocardium, ferroptosis and pyroptosis primarily affect cardiomyocytes during ischemia–reperfusion injury and post-infarction remodeling, resulting in contractile-cell loss and fibrotic replacement (Liu D. et al., 2024; Mu et al., 2025; Shen et al., 2025c; Zhou et al., 2021); these compartment-specific assignments are largely based on marker expression and inhibitor pharmacology, and few studies have applied the genetic rescue criteria outlined in the Introduction.

### 5.3 Gut-lung axis and acute respiratory distress syndrome

The gut-lung axis represents a critical bidirectional communication pathway whereby alterations in the gut microbiota can profoundly influence pulmonary health and disease, including the pathogenesis and progression of ARDS. ARDS is characterized by severe pulmonary inflammation, alveolar epithelial and endothelial damage, and hypoxemic respiratory failure, with high morbidity and mortality rates. Emerging evidence indicates that dysbiosis of the gut microbiota contributes to ARDS development by modulating systemic and pulmonary immune responses, barrier integrity, and inflammatory cascades. Genetic studies using Mendelian randomization have suggested associations between specific gut microbial taxa—such as Streptococcus, Bifidobacterium, and Akkermansia—and the risk or severity of ARDS, although causality remains to be definitively established (Huang et al., 2025; Li C. et al., 2025). Experimental models demonstrate that disruption of gut microbiota, for example via broad-spectrum antibiotics, exacerbates lung inflammation induced by LPS, highlighting the protective role of a balanced gut microbiome in acute lung injury (ALI) and ARDS (Jacobs et al., 2020).

Mechanistically, gut microbiota alterations influence pulmonary epithelial and immune cell death pathways, including pyroptosis and ferroptosis, which are pivotal in ARDS pathophysiology; alveolar epithelial cells are a principal target, and their dysfunction is a recognized driver of barrier failure in ARDS (Wang and Chao, 2025). Dysbiosis-induced intestinal barrier dysfunction facilitates translocation of microbial products and endotoxins into systemic circulation, triggering alveolar macrophage activation, neutrophil extracellular trap formation, and an imbalance in regulatory T cells (Tregs) and T helper 17 (Th17) cells in the lung microenvironment (Li Q. et al., 2025; Ziaka and Exadaktylos, 2024). This cascade exacerbates oxidative stress, inflammatory cell death, and tissue damage in the lungs. Moreover, pulmonary inflammation reciprocally impacts gut barrier integrity and microbiota composition, creating a vicious cycle that perpetuates systemic inflammation and organ dysfunction (Li Q. et al., 2025). Innovative therapies employing butyric acid-loaded nanoparticles have demonstrated efficacy in restoring gut-lung axis homeostasis and attenuating inflammation via modulation of key signaling pathways like phosphoinositide 3-kinase-protein kinase B signaling pathway (PI3K-AKT) and protein tyrosine phosphatase nonreceptor type 1 (PTPN1) (Zhu et al., 2025). Similarly, probiotic strains such as *Lactobacillus fermentum* have been shown to remodel lung microbiota and mitigate LPS-induced lung injury through gut-lung crosstalk (Ni et al., 2026). Because butyrate-loaded nanoparticles and orally administered probiotics both act systemically as well as through the microbiota, these interventions demonstrate therapeutic benefit but do not by themselves establish a microbiota-dependent mechanism.

In the context of viral infections such as COVID-19, gut microbiota dysbiosis correlates with gastrointestinal symptoms and persistent inflammation, contributing to ARDS and post-acute sequelae through disrupted mucosal immunity and altered microbial communities (Fanelli et al., 2022; Moon, 2023). Nutritional interventions, including vitamin D supplementation and the use of probiotics and prebiotics, may support gut microbiota balance and attenuate hyperinflammatory states, potentially reducing ARDS severity (Batista et al., 2023; Nithin et al., 2021). Furthermore, endogenous lipid mediators like anandamide have been found to attenuate ARDS by modulating the gut-lung microbiome axis, inducing antimicrobial peptides, tight junction proteins, and SCFA production, thereby stabilizing immune homeostasis (Sultan M. et al., 2021).

### 5.4 Microbial-cell death interactions in liver diseases

The gut–liver axis differs from other organ axes because microbial products and metabolites reach the liver directly through the portal circulation. Consequently, Kupffer cells and hepatocytes represent two mechanistically distinct targets. Microbial products and altered bile-acid signaling primarily promote inflammasome activation and pyroptosis in Kupffer cells, whereas mitochondrial dysfunction, iron accumulation, and phospholipid peroxidation increase ferroptotic susceptibility in hepatocytes. In MASLD, dysbiosis-associated changes in bile-acid pools and intestinal permeability intersect with hepatic lipid accumulation and mitochondrial dysfunction. In Kupffer cells, microbial-product sensing can reinforce NLRP3-dependent inflammatory signaling; in hepatocytes, impaired fatty-acid oxidation and reduced antioxidant capacity promote lipid peroxidation and ferroptosis. This division helps distinguish inflammatory immune-cell death from metabolic parenchymal-cell injury.

Heavy metal exposure, including cadmium, lead, arsenic, and mercury, has been increasingly implicated in the pathogenesis of MASLD and its inflammatory subtype metabolic dysfunction-associated steatohepatitis (MASH; formerly non-alcoholic steatohepatitis, NASH). Mechanistically, heavy metals induce hepatic lipid accumulation through upregulation of lipogenic transcription factors such as SREBP-1, ChREBP, and PPARγ, alongside downregulation of fatty acid β-oxidation regulators like PPARα. This dysregulation leads to oxidative stress, endoplasmic reticulum stress, and activation of RCD pathways, notably pyroptosis and ferroptosis, which contribute to hepatocyte injury and inflammation. These findings underscore a complex interplay where heavy metal-induced gut dysbiosis and microbial metabolites modulate ferroptotic pathways in the liver, amplifying liver injury. Understanding these interactions may reveal novel therapeutic targets to mitigate heavy metal-associated liver diseases by modulating gut microbiota and ferroptosis pathways (Shao et al., 2024; Tinkov et al., 2023).

**Figure 4:**
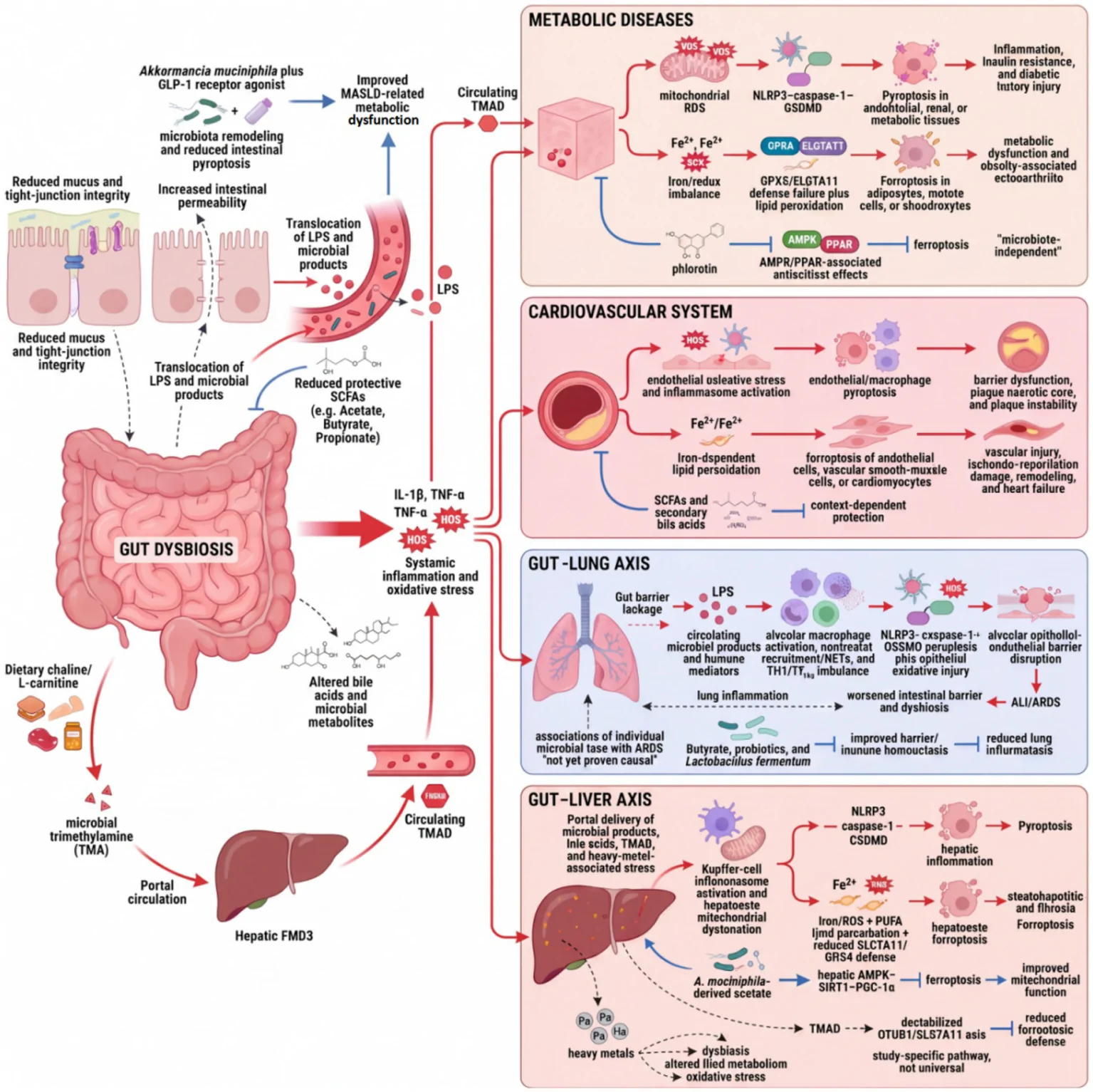
Organ-specific regulated cell death driven by gut dysbiosis. Intestinal barrier dysfunction and microbial-product leakage represent shared upstream events, whereas circulation routes, target cells, microbial metabolites, and local metabolic conditions determine organ-specific cell-death outcomes. In metabolic tissues and the kidney, dysbiosis-associated inflammation, TMAO, mitochondrial ROS, and iron-redox imbalance promote pyroptotic and ferroptotic injury. In the cardiovascular system, endothelial and macrophage pyroptosis and ferroptosis of vascular or myocardial cells contribute to atherosclerosis, ischemia–reperfusion injury, remodeling, and heart failure. In the gut–lung axis, microbial translocation and immune dysregulation promote alveolar macrophage pyroptosis and epithelial–endothelial injury in ALI/ARDS. In the gut–liver axis, portal microbial signals, altered bile acids, TMAO, and toxicant-associated stress drive Kupffer-cell inflammasome activation and hepatocyte ferroptosis, contributing to MASLD/MASH and fibrosis.

Mitochondrial dysfunction is a central feature in MASLD pathogenesis, contributing to impaired fatty acid oxidation, increased ROS generation, and lipid peroxidation. These mitochondrial perturbations trigger RCD pathways, including pyroptosis and ferroptosis, which exacerbate hepatic inflammation and fibrosis. Gut microbiota modulates ferroptosis through multiple mechanisms: by influencing intestinal barrier integrity and bacterial translocation, altering bile acid profiles that regulate hepatic lipid metabolism, and producing metabolites such as SCFAs that impact oxidative stress responses. For example, beneficial microbes like *Akkermansia muciniphila* produce acetate, which activates the hepatic AMPK/SIRT1/PGC-1α axis, enhancing mitochondrial function and inhibiting ferroptosis. Conversely, gut dysbiosis leads to increased levels of deleterious metabolites like TMAO, which promotes ferroptosis via destabilization of the OTUB1/SLC7A11 axis in hepatocytes. Therapeutic interventions targeting the gut microbiota-ferroptosis axis, including probiotics, prebiotics, and natural compounds such as ginsenoside Rd. and ursolic acid, have demonstrated efficacy in ameliorating mitochondrial dysfunction, suppressing pyroptosis and ferroptosis, and improving MASLD outcomes. Moreover, mitochondrial bioenergetics alterations influence pyroptotic cell death in hepatic macrophages, linking metabolic dysfunction to immune activation. Collectively, these insights highlight the gut-liver axis as a critical regulator of mitochondrial function and RCD in MASLD, offering promising avenues for precision therapies that modulate microbial composition and cell death pathways to halt disease progression (Drummer et al., 2023; Gao et al., 2026; Ji et al., 2023; Liu W. et al., 2025; Termite et al., 2025; Zhuge et al., 2025; Figure 4).

## 6 Targeting gut microbiota to regulate pyroptosis and ferroptosis therapeutic strategies

### 6.1 TCM and natural products application

TCM and natural products have garnered increasing attention for their multifaceted roles in modulating gut microbiota and regulating RCD pathways such as pyroptosis and ferroptosis, which are critical in various diseases. A recurring interpretive problem in this literature is that most natural products exert direct pharmacological effects on host cell-death machinery in addition to, or instead of, acting through the microbiota. Baicalin, ginsenoside Rd., paeoniflorin and xanthohumol all inhibit lipid peroxidation or inflammasome assembly in purely *in vitro* systems, so a microbiota-dependent mechanism cannot be inferred merely because microbial composition also changes after treatment. Attribution to the microbiota requires at least one of the following: loss of efficacy in germ-free or antibiotic-treated animals, transfer of the therapeutic effect by fecal transplantation from treated to untreated recipients, or reproduction of the effect by a defined microbial metabolite at physiologically achievable concentrations. Few of the studies cited below satisfy these criteria. Throughout this section, therefore, terms such as “microbiota-mediated” are reserved for interventions meeting at least one of them, and the remaining effects are described as occurring in the presence of, rather than because of, microbial remodeling. Purified monomers are generally more suitable for mechanism-oriented studies, as their defined structures allow clearer linkage between microbial remodeling, host metabolites, and cell-death molecular endpoints. For example, paeoniflorin was reported to alleviate diabetic cardiomyopathy by reshaping gut microbiota and microbial metabolites, with 11,12-epoxyeicosatrienoic acid serving as a potential mediator of its anti-ferroptotic effect (Wu et al., 2024). Similarly, ginsenoside Rd. was shown to modulate the gut microbiome and lipid peroxidation-mediated ferroptosis pathway in metabolic dysfunction-associated fatty liver disease, supporting a gut–liver ferroptosis mechanism (Liu W. et al., 2025). Compared with purified monomers, plant extracts or enriched fractions usually exert broader metabolic effects, including changes in SCFA production, bile acid transformation, oxidative stress buffering, and epithelial repair, but their active constituents and dose–exposure relationships are often more difficult to define. Compound TCM formulas are even more complex because they may simultaneously regulate microbiota composition, epithelial barrier function, immune-cell polarization, and inflammatory or ferroptotic thresholds. This feature distinguishes TCM-derived interventions from synthetic drugs. Synthetic inhibitors are usually designed to target a defined molecular node, such as NLRP3, GPX4, ACSL4, or SLC7A11, whereas TCM-derived strategies may provide broader microbiota–host network remodeling but face greater challenges in chemical standardization, pharmacokinetic characterization, reproducibility, and causal attribution.

The multi-target therapeutic potential of TCM extends notably to chronic diseases such as CVDs, diabetes mellitus, and cancer. In cardiovascular disease models, TCM formulations and their bioactive constituents have been shown to regulate gut microbiota composition, reduce pro-inflammatory metabolites like TMAO, and enhance SCFA production, collectively mitigating pyroptosis and ferroptosis-mediated vascular injury and atherosclerosis progression (Cheng et al., 2021; Deng et al., 2022; Fan et al., 2025). In DM, natural products derived from TCM modulate gut microbiota to restore metabolic balance, reduce oxidative stress, and inhibit ferroptosis in pancreatic β-cells and target organs, improving insulin sensitivity and preventing diabetic complications (Jiang et al., 2022; Niazpour and Meshkani, 2025). Moreover, TCM’s capacity to regulate RCD pathways has been implicated in cancer therapy, where natural compounds induce ferroptosis and pyroptosis in tumor cells while modulating immune checkpoints such as PD-1/PD-L1, enhancing antitumor immunity (Ding et al., 2026; Shi et al., 2025; Xie et al., 2023; Zheng et al., 2023).

The interplay between TCM, gut microbiota, and RCD also underlies therapeutic effects in neurodegenerative diseases, IBD, and metabolic disorders. For example, TCM polysaccharides are metabolized by gut microbiota into bioactive SCFAs, which suppress neuroinflammation and pyroptosis in neurodegenerative models (Pasupalak et al., 2024; Yue et al., 2022). In ulcerative colitis, TCM modulates gut microbiota to inhibit pyroptosis and ferroptosis of IECs, promoting mucosal healing and reducing inflammation (Feng et al., 2022; Wang Y. et al., 2024; Yuan et al., 2025). Additionally, TCM interventions have shown promise in obesity and metabolic syndrome by restoring gut microbial balance and inhibiting RCD pathways that contribute to metabolic inflammation (Li et al., 2023; Li et al., 2021).

### 6.2 Microbiota modulators and metabolite interventions

The modulation of gut microbiota through probiotics, SCFAs, and FMT represents a promising strategy to regulate RCD-related diseases by restoring intestinal microbial homeostasis and influencing host metabolic and immune pathways. The interventions in this section differ in how securely their effects can be attributed to the microbiota. FMT and defined-strain colonization act on the microbial community by definition and therefore provide level 2 evidence when paired with a cell-death readout. Orally administered SCFAs, bile acids and tryptophan derivatives, by contrast, are absorbed and act directly on host receptors, so their effects are pharmacological unless the same outcome is reproduced by manipulating endogenous microbial production. Probiotics, such as *Lactobacillus kefiranofaciens* ZW18, have demonstrated efficacy in enhancing antitumor immune responses when combined with immune checkpoint inhibitors like PD-1 blockers, by promoting CD8 + T cell infiltration and reshaping gut microbiota composition, including increasing beneficial taxa like Akkermansia and Prevotellaceae (Zhao et al., 2022). Similarly, SCFAs, primarily produced by bacterial fermentation of dietary fibers, exert multifaceted beneficial effects including improving gut barrier integrity, modulating glucose and lipid metabolism, and regulating immune responses and inflammation, which collectively contribute to the amelioration of cardio-metabolic disorders and inflammatory diseases (Nie et al., 2025; Nogal et al., 2021). Dietary interventions that increase SCFA production, such as supplementation with chitin-glucan or alginate oligosaccharides, have been shown to beneficially alter gut microbiota composition and metabolite profiles, thereby improving metabolic syndrome parameters and reducing inflammation (Rodriguez et al., 2020; Wang J. et al., 2024). FMT has also emerged as a therapeutic modality to restore microbial diversity and function, with evidence supporting its role in modulating immune responses and metabolic pathways in diseases such as T2D and IBD (He et al., 2025; Liu et al., 2025b). Targeted metabolite therapies focusing on microbial metabolites, including bile acids and tryptophan derivatives, offer additional avenues for intervention. Microbiota-derived bile acid metabolic enzymes influence host metabolism and immune homeostasis, and their dysregulation is implicated in metabolic and inflammatory disorders, suggesting that manipulating bile acid profiles could yield therapeutic benefits (Ma H. et al., 2025). Moreover, the modulation of tryptophan metabolism by gut microbes affects neuroinflammation and depressive disorders, highlighting the potential of metabolite-targeted strategies in neuropsychiatric conditions (Du et al., 2025). The gut microbiota also mediates the circadian regulation of host metabolism through extracellular vesicles, underscoring the complexity of host-microbe metabolic crosstalk (Tan et al., 2025). Engineered and synthetic-biology approaches to restoring microbial balance are also being developed and may eventually allow more defined manipulation of these pathways (Mkilima, 2025). Collectively, these findings underscore the therapeutic potential of microbiota modulators and their metabolic products in alleviating diseases linked to RCD pathways by restoring microbial balance, enhancing beneficial metabolite production, and modulating host immune and metabolic functions. Future research integrating multi-omics and clinical trials is essential to optimize these interventions for precision medicine applications.

### 6.3 Emerging technologies and precision medicine strategies

Multi-omics profiling combined with artificial intelligence (AI) is increasingly used to map gut microbiota–host interactions relevant to pyroptosis and ferroptosis, and is presented here as an enabling research direction rather than as an established mechanism. Multi-omics technologies—encompassing genomics, transcriptomics, proteomics, metabolomics, and epigenomics—enable comprehensive profiling of microbial communities and host responses at multiple biological layers, and single-cell approaches have already resolved epithelial and immune heterogeneity in IBD (Corridoni et al., 2020). Analyzed with machine-learning and deep-learning methods, such datasets can prioritize candidate microbial species, enzymes and metabolites that plausibly modulate ferroptosis through iron metabolism and lipid peroxidation, or pyroptosis through inflammasome activation and GSDM cleavage (Kharb and Zhu, 2026; Zhang C. et al., 2025), and may support biomarker discovery and patient stratification in IBD, colorectal cancer and metabolic disorders (Aldriwesh et al., 2026; Bonazzi et al., 2025). However, multi-omics and AI identify correlations rather than causal mechanisms, and model performance is strongly affected by cohort size, batch effects, diet, medication exposure and population heterogeneity. To date, no AI-prioritized microbe–metabolite–cell-death axis has been experimentally validated end to end. These approaches should therefore be regarded as hypothesis-generating tools whose outputs require the genetic and gnotobiotic validation described in the Introduction.

Gut organoids, microfluidic gut-on-chip systems and spatial omics are complementary experimental platforms that could close this validation gap, although their application to microbiota-regulated cell death is still at an early stage. Anaerobic intestine-on-a-chip devices sustain complex human gut communities in contact with living epithelium under physiological oxygen gradients and flow, permitting controlled co-culture with defined microbes and immune cells (Jalili-Firoozinezhad et al., 2019). Such systems are, in principle, well suited to real-time monitoring of pyroptosis and ferroptosis under defined microbial exposure, and to the loss-of-function tests that observational studies cannot provide, although they do not yet reproduce systemic immunity, innervation or the gut–organ axes discussed in section 2.4. In parallel, spatial technologies now allow host transcriptomes and bacterial 16S sequences to be captured simultaneously from intact tissue, resolving microbial niches alongside host responses (Lötstedt et al., 2023). Applied to inflamed or tumor tissue, these methods could identify microenvironmental niches in which dysbiosis coincides with pyroptotic or ferroptotic activity, but the necessary studies have not yet been performed, and current spatial resolution and transcript capture efficiency remain limiting. These platforms are therefore best regarded as future research directions for causal dissection rather than as sources of established mechanism.

Gene editing and nanocarrier-based delivery are likewise presented here as prospective tools rather than as demonstrated therapies for microbiota-regulated cell death. Genome-scale CRISPR-Cas9 screening is an established route to functional validation of host death pathways and was central to the original identification of GSDMD as the pyroptotic executioner (Shalem et al., 2014; Shi et al., 2015); the same approach can be applied to inflammasome components, GPX4 and iron-metabolism regulators, and has been used to define ferroptotic vulnerabilities in tumor cells (Yin et al., 2024). Its use to test whether specific microbial signals require particular host genes to trigger cell death is, however, largely unexplored. For *in vivo* delivery, engineered nanocarriers including liposomes, metal–organic frameworks and polymeric nanoparticles offer targeted transport of editing components or small-molecule modulators to IECs or immune cells, with stimuli-responsive and controlled-release designs improving precision and limiting off-target exposure (Mitchell et al., 2021; Shan et al., 2020). Proof-of-concept examples include ferroptosis-driven nanotherapeutics and metal–organic frameworks designed for ferroptosis-mediated cancer therapy (Ma et al., 2026; Shan et al., 2020), and liposomal delivery of 7-dehydrocholesterol to modulate radiation-induced ferroptosis (Li J. et al., 2025). None of these platforms has yet been used to manipulate a defined microbiota–cell-death axis in vivo, and their translation will require evidence on biodistribution, immunogenicity, microbiota perturbation by the carrier itself, and long-term safety.

### 6.4 Future challenges and research directions

Although microbiota-targeted strategies provide a promising route for modulating RCD, their clinical translation faces challenges that are more specific than low bioavailability or inter-individual variation alone. One major issue is the safety and controllability of FMT. FMT is effective for recurrent *Clostridioides difficile* infection, but its broader application to inflammatory, metabolic, or cancer-related RCD remains less established. Reported adverse events include infections and other serious complications, and regulatory agencies have warned that FMT products may transmit pathogenic or multidrug-resistant organisms if donor screening is insufficient (Kelly et al., 2020; Wang et al., 2016). Therefore, future FMT-related studies in microbiota-regulated RCD should move beyond global microbiota replacement and incorporate standardized donor screening, pathogen surveillance, strain-level tracking, metabolomic monitoring, and long-term follow-up.

Recent clinical studies also suggest that microbiota modulation is beginning to move from supportive care toward combination therapy, particularly in cancer immunotherapy. In a multicenter phase I trial, healthy-donor FMT combined with nivolumab or pembrolizumab was tested in 20 previously untreated patients with advanced melanoma, supporting the feasibility of integrating microbiota intervention with anti-PD-1 therapy, although larger randomized studies are still required (Routy et al., 2023). More recently, the phase 2 FMT-LUMINate trial evaluated oral-capsule FMT combined with immune checkpoint blockade in first-line NSCLC and melanoma (Duttagupta et al., 2026), further indicating that microbiota-based interventions are entering prospective oncology trials, but their optimal donor selection, engraftment durability, immune correlates, and cell-death-related biomarkers remain unresolved; notably, in that trial clinical response was associated with loss of baseline commensal species rather than with donor engraftment, illustrating how difficult mechanistic attribution remains even in randomized settings. In parallel, standardized live biotherapeutic products, such as SER-109, an oral consortium of purified Firmicutes spores that reduced recurrence of Clostridioides difficile infection compared with placebo in a randomized trial, illustrate a more controlled alternative to conventional FMT and may provide a regulatory model for future microbiota-based interventions (Feuerstadt et al., 2022).

Another challenge is the gap between animal models and human disease. Many microbiota–RCD mechanisms are discovered in germ-free, antibiotic-treated, or chemically induced mouse models, but these systems do not fully reproduce human diet, medication exposure, microbial ecology, immune history, disease chronicity, or inter-organ communication (Van Hul and Cani, 2026). This is particularly relevant for RCD studies, because inflammasome priming, ferroptotic sensitivity, mitochondrial metabolism, and immune-cell polarization are all strongly influenced by host species, tissue context, and environmental exposure. Thus, promising findings in animal models should be validated through humanized microbiota models, patient-derived organoids, gut-on-chip systems, longitudinal clinical sampling, and multi-omics integration before being interpreted as therapeutic mechanisms.

**Figure 5:**
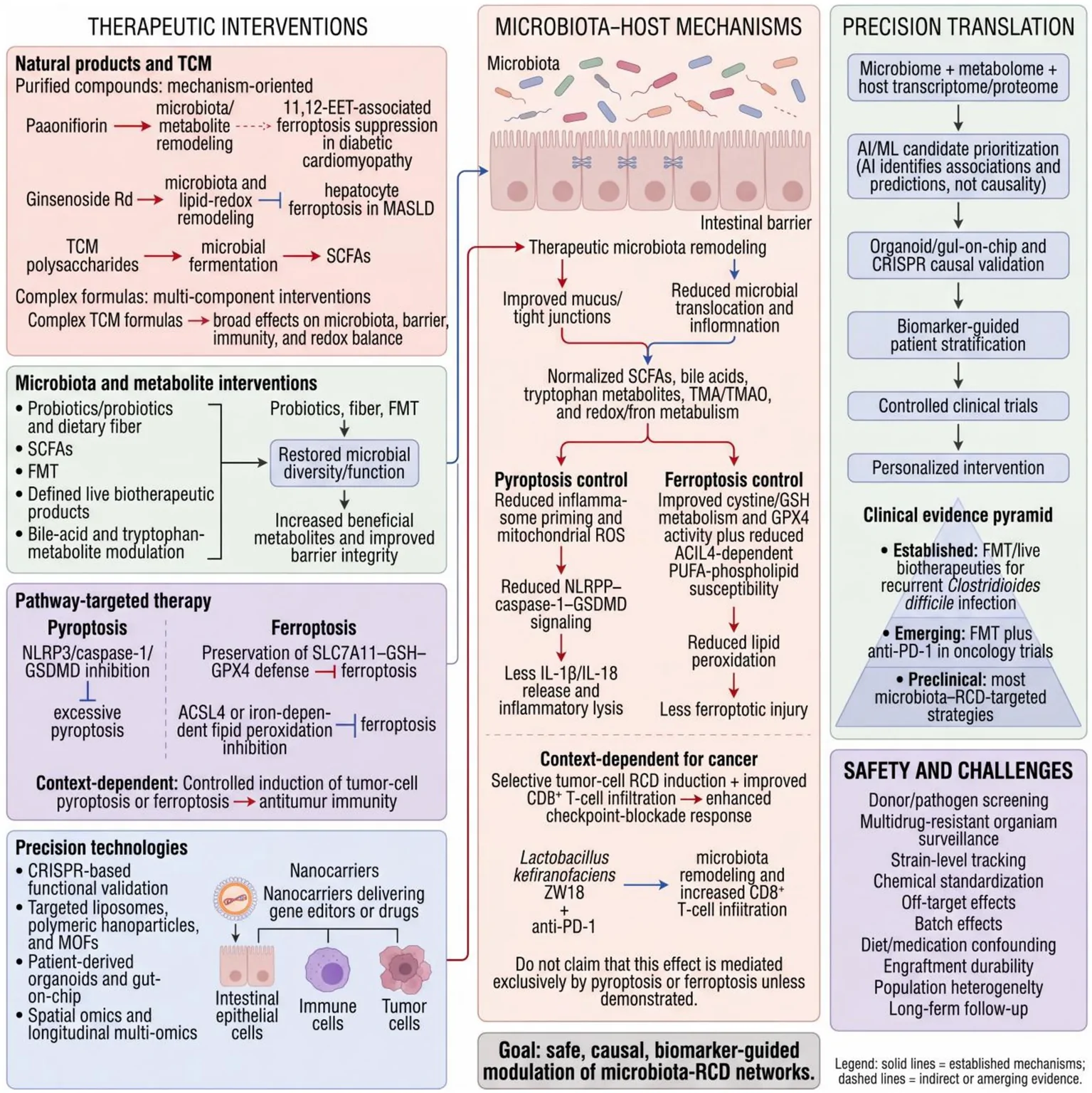
Therapeutic strategies targeting microbiota-regulated cell death. Natural products and TCM preparations, probiotics, prebiotics, dietary interventions, SCFAs, FMT, defined live biotherapeutics, and metabolite-directed therapies may restore microbial homeostasis, strengthen the intestinal barrier, and regulate inflammatory and redox pathways. These interventions can suppress excessive NLRP3–caspase-1–GSDMD pyroptosis or preserve SLC7A11–GSH–GPX4-dependent ferroptosis resistance; conversely, selective induction of regulated cell death in tumor cells may enhance antitumor immunity. Multi-omics, artificial intelligence, spatial profiling, patient-derived organoids, gut-on-chip systems, CRISPR-based validation, and targeted nanodelivery provide a framework for causal discovery and personalized treatment. Clinical translation requires standardized products, pathogen surveillance, biomarker-guided stratification, mechanistic validation, and rigorous long-term safety and efficacy assessment.

Furthermore, the heterogeneity in patient microbiomes and immune status necessitates personalized therapeutic strategies. Recent studies highlight that microbial strain-level differences critically influence responses to immune checkpoint blockade therapies, underscoring the need for precise microbiome diagnostics tailored to treatment regimens (Gunjur et al., 2024). Additionally, the modulation of RCD pathways by microbiota-derived metabolites varies among individuals, influenced by diet, genetics, and comorbidities, complicating the prediction of therapeutic outcomes (Wang Z. et al., 2025; Zhou J. et al., 2025). Therefore, integrating microbiome profiling with biomarker development and leveraging machine learning for patient stratification are essential future directions. Rigorous clinical trials with standardized protocols and long-term follow-up are imperative to establish the safety and efficacy of these interventions. Ultimately, overcoming these challenges will pave the way for microbiota-based personalized medicine that effectively harnesses the regulation of pyroptosis, ferroptosis, and related mechanisms for disease treatment and prevention (Figure 5).

## 7 Conclusion

In conclusion, the emerging interface between the gut microbiota and RCD provides a unifying framework for understanding how microbial signals are translated into tissue-specific inflammatory and metabolic injury. Microbial components and metabolites can influence pyroptosis and ferroptosis by engaging innate immune sensors, reshaping redox and iron homeostasis, altering epithelial barrier integrity, and modifying systemic immune and metabolic states. However, these effects cannot be reduced to a simple model in which dysbiosis uniformly promotes cell death. Their biological consequences depend on the identity of the microbial signal, its concentration and route of exposure, the responding cell population, and the stage and tissue context of disease. Pyroptosis may support antimicrobial defense when spatially and temporally controlled but amplify epithelial or immune-mediated injury when excessive; similarly, ferroptosis may contribute to parenchymal damage in metabolic, cardiovascular, and neurological disorders while representing a potentially exploitable vulnerability in tumor cells. Importantly, although several microbial metabolites and host receptors have been linked directly to cell-death machinery, many reported microbiota–cell death relationships remain associative or are mediated indirectly through inflammation, barrier dysfunction, and systemic metabolic remodeling. Applying the four-level evidence scheme introduced above, only a minority of the relationships surveyed here reach level 1 or level 2, and cuproptosis and disulfidptosis remain at level 4 throughout.

The next phase of research should move beyond parallel measurements of microbial composition and cell-death markers toward experiments that establish directionality, cellular origin, and molecular necessity. In human studies, longitudinal sampling combined with metagenomics, metabolomics, immune profiling, and tissue-based assessment of regulated cell death will be essential to determine whether microbial alterations precede disease progression, modify its trajectory, or simply reflect established pathology. These principles are equally important for evaluating probiotics, dietary interventions, natural products, and microbial metabolites, whose effects may arise from direct pharmacological activity rather than microbiota-dependent mechanisms. Thus, the translational value of this field will depend less on broadly “targeting the microbiota” than on identifying reproducible microbial functions, responsive host-cell populations, and disease-specific therapeutic windows. Such mechanistic precision may ultimately enable microbiota-informed modulation of regulated cell death, but its clinical application will require rigorous validation, standardized methodologies, and carefully stratified human studies.
